# Model-Free or Not?

**DOI:** 10.3389/fmolb.2021.727553

**Published:** 2021-10-25

**Authors:** Kai Zumpfe, Albert A. Smith

**Affiliations:** Institute for Medical Physics and Biophysics, Medical Faculty, Leipzig University, Leipzig, Germany

**Keywords:** solid-state NMR, dynamics detectors, model-free analysis, NMR relaxation, molecular dynamics simulation

## Abstract

Relaxation in nuclear magnetic resonance is a powerful method for obtaining spatially resolved, timescale-specific dynamics information about molecular systems. However, dynamics in biomolecular systems are generally too complex to be fully characterized based on NMR data alone. This is a familiar problem, addressed by the Lipari-Szabo model-free analysis, a method that captures the full information content of NMR relaxation data in case all internal motion of a molecule in solution is sufficiently fast. We investigate model-free analysis, as well as several other approaches, and find that model-free, spectral density mapping, LeMaster’s approach, and our *detector* analysis form a class of analysis methods, for which behavior of the fitted parameters has a well-defined relationship to the distribution of correlation times of motion, independent of the specific form of that distribution. In a sense, they are all “model-free.” Of these methods, only detectors are generally applicable to solid-state NMR relaxation data. We further discuss how detectors may be used for comparison of experimental data to data extracted from molecular dynamics simulation, and how simulation may be used to extract details of the dynamics that are not accessible via NMR, where detector analysis can be used to connect those details to experiments. We expect that combined methodology can eventually provide enough insight into complex dynamics to provide highly accurate models of motion, thus lending deeper insight into the nature of biomolecular dynamics.

## Introduction

Study of biomolecular function requires understanding the dynamics of the biological system. Nuclear magnetic resonance (NMR), despite many recent technological advances in other techniques, remains a premier method for detailed dynamics characterization. In NMR, one may measure a variety of site-specific relaxation experiments, which provide timescale sensitive information about the motion. By varying the type of experiment (*T*_1_, *T*_1*ρ*_, NOE, etc.) or experimental conditions (external magnetic field, applied field strength, magic-angle spinning (MAS) frequency, etc.), the timescale sensitivity of the measurement is modified. Then, one may resolve the dynamics both in space, via site resolution, and in timescale, via multiple experiments (Palmer, 2004; Schanda and Ernst, 2016).

However, is it possible to fully characterize the motions leading to the observed relaxation behavior? Many relaxation experiments in NMR are sensitive to the reorientational motion of anisotropic NMR interaction tensors (NMR relaxation can also be sensitive to change in scalar terms, e.g., isotropic chemical shift). For a given spin, relaxation is usually dominated by only one to two interactions. For example, relaxation of ^15^N in a protein backbone is determined almost entirely by the reorientation of the one-bond ^1^H–^15^N dipole coupling and the ^15^N chemical shift anisotropy (CSA). But, multiple sources of motion lead to reorientation of the bond. For example, if we suppose the H–N bond to be in a protein, within a helix, then we would have local distortion of the peptide plane (one-bond libration), motion of the peptide plane within the helix, motion of the helix within the protein, and motion of the protein either in solution, in a crystal, a fibril, a membrane, etc.

**FIGURE 1:**
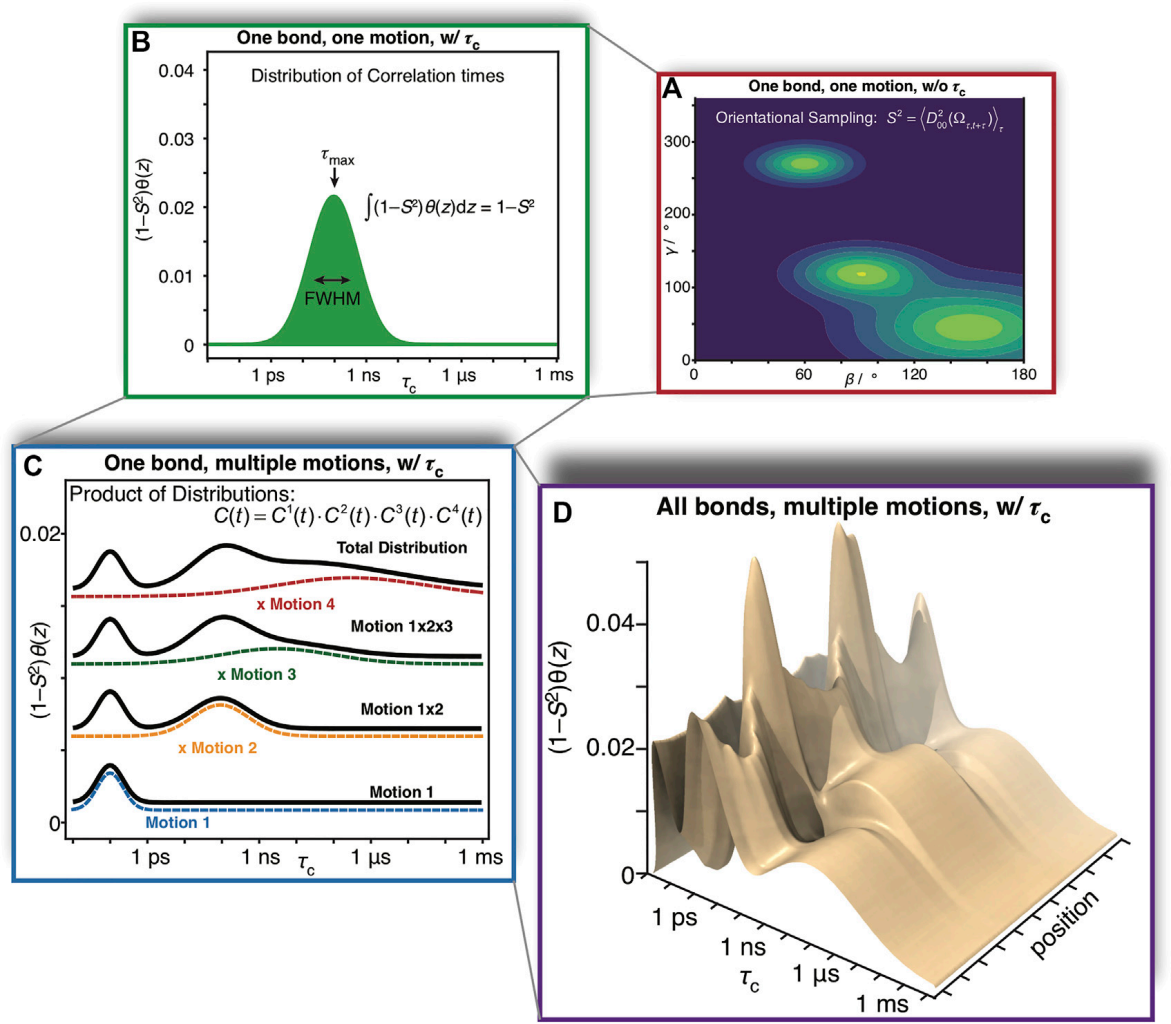
Complexity of reorientational dynamics. For each bond in a molecule, multiple types of motion result in orientational sampling, where the distribution of angles for each motion result in a generalized order parameter, *S* ^2^. Therefore, in **(A)** we plot a possible distribution of Euler angles for a single type of motion (population is plotted as a function of angles *β* and *γ*, where *α* is not required for a symmetric interaction tensor). A single motion is furthermore described by a correlation time, and may be distributed over a range of correlation times. In **(B)** we plot a possible distribution of correlation times $\left(1-S^{2}\right)\theta \left(z\right)$ , that is, amplitude of motion as a function of the log-correlation time, $z=\mathrm{lo}g_{10}\left(\tau_{c}/s\right)$ . Each distribution is characterized by an amplitude, center, and width. Note that the integral of the distribution is $\left(1-S^{2}\right)$ , *S* ^2^ being determined by the distribution of angles in **(A)**. While **(A,B)** illustrate aspects of a single motion, multiple motions influence a given bond, where the total correlation function is the product of individual correlation functions. In **(C)**, we plot four distributions of motion (color). Above each motion, we plot the distribution resulting from the product of that motion and all motions below it (black), eventually resulting in the total distribution seen at the top. Finally, we note that the total distribution varies as a function of position in the molecule, resulting in the 3D plot of the distribution as a function of correlation time and position in the molecule observed in **(D)**. While this is just an illustration, one could imagine that motion in **(D)** results from three α-helices in a protein, each having a slightly different behavior, and varying dynamics as one approaches the end of each helix.

This degree of complexity is illustrated in Figure 1. For a given bond in a molecule, and a given motion acting on that bond, a distribution of orientations is sampled as illustrated in Figure 1A. The orientational distribution determines the contribution of that motion to the total order parameter, $S^{2}$ . However, not only are there many orientations sampled by a bond due to a motion, but those orientations are sampled at some rate, such that the motion has an associated correlation time or distribution of correlation times (denoted $\left(1-S^{2}\right)\theta \left(z\right)$ ). We illustrate this in Figure 1B; note that not only is the width of such a distribution variable, but also the functional form of the distribution itself. This results in a correlation function that decays from 1 to $S^{2}$ , where integrating over the distribution of correlation times yields the total amplitude of the decay. Already, a single bond with just one motion acting on it yields potentially a high degree of complexity; however, we must still consider that multiple motions act on each bond, where the total correlation function is the product of the correlation functions of each individual motion (if those motions are independent from one another, Figure 1C). Finally, motion varies throughout a molecule, as a function of position, resulting in a complex, multi-dimensional description as illustrated in Figure 1D.

While NMR is powerful, obtaining a complete description of the complex dynamics stretches beyond the limit of what is possible based on experimental data alone, especially for large molecules such as proteins. This problem is a familiar one, addressed almost 40 years ago by Lipari and Szabo (Lipari and Szabo, 1982a), who developed a method known as the *model-free* approach. While we will discuss the details of this approach below, the name tells us a critical advantage of such an approach: model-free analysis allows the extraction of dynamics information from NMR relaxation data *without having knowledge of the specific model of motion.* Furthermore, the resulting parameters have a well-defined relationship to the distribution of orientations sampled and the distribution of correlation times.

Lipari and Szabo described the internal motion of a molecule with just two parameters: a generalized order parameter related to the amplitude of motion, $S^{2}$ , and a mean effective correlation time, $\langle \tau_{e}\rangle$ (a third parameter, $\tau_{\text{M}}$ , gives the correlation time of the molecule tumbling in solution). While only two parameters suggests a simple analysis, it is important to note that Lipari and Szabo did not intend to only describe simple motions having just a single correlation time and amplitude: theoretical tests of their model were performed on a wobbling-on-a-cone model (Kinosita et al., 1977) that results in a weighted sum of correlation times, and experimental work was performed on methyl groups in a protein, for which the total motion is determined by the product of methyl rotation and by reorientation of the methyl group’s C–C bond. Rather, the two parameters contain the aggregated information describing all motions that is available from the set of relaxation experiments alone.

**FIGURE 2:**
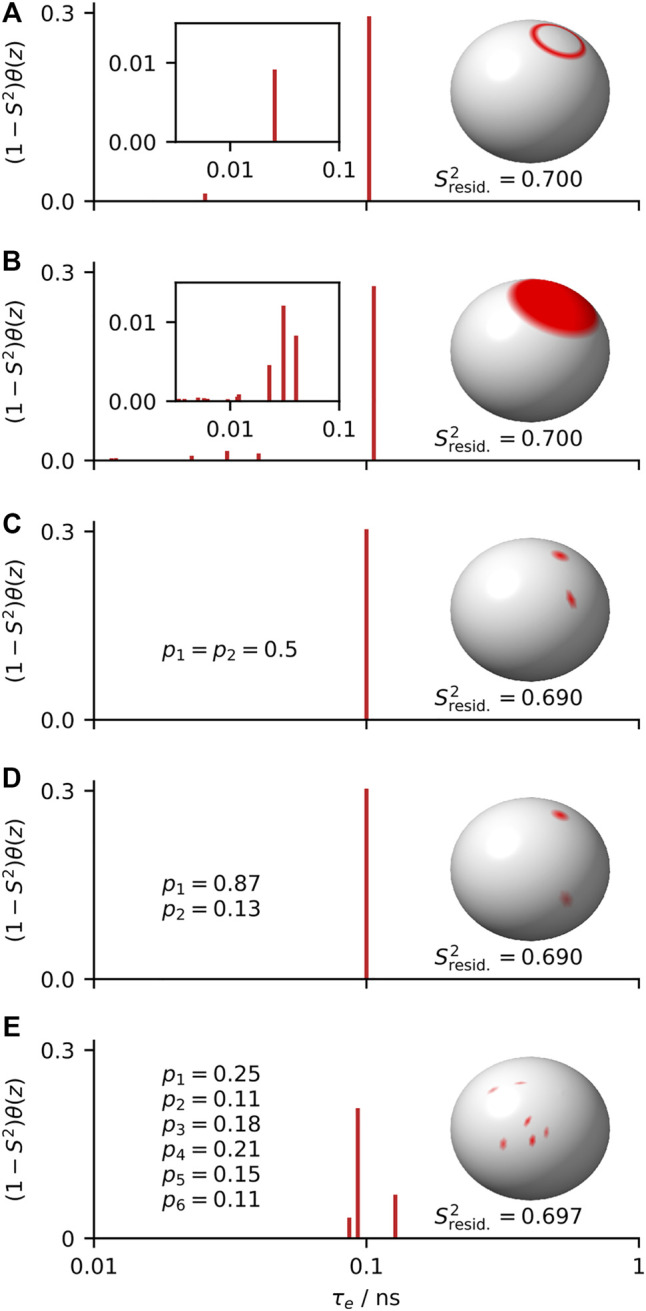
Five distributions of orientations and correlation times that yield the same model-free parameters ( $\left(1-S^{2}\right)$ = 0.3, $\langle \tau_{e}\rangle$ = 0.1 ns). In **(A–E)**, we plot a distribution of orientations (sphere, right); on the axes, we plot the distribution of correlation times resulting from exchange among that set of orientations. Models of motion are wobbling-on-a-cone ( $\theta_{\text{cone}}=19^{{}^{\circ}}$ ), wobbling-in-a-cone ( $\theta_{\mathrm{cone}}=28^{{}^{\circ}}$ ), symmetric two-site hop ( $\theta_{\mathrm{hop}}=39^{{}^{\circ}}$ ), asymmetric two-site hop ( $\theta_{\mathrm{hop}}=70^{{}^{\circ}}$ ), and 6-site asymmetric exchange. Insets in **(A,B)** show correlation times with small amplitudes. $S_{\mathrm{resid}.}^{2}$ refers to the order parameter from residual couplings (see Supplementary Section S3), which deviates from the generalized order parameter for asymmetric motion.

The advantage of model-free analysis is that it does not require knowing the model of motion. For example, for relatively low fields (∼90 MHz, as used by Lipari and Szabo), all distributions of orientations and correlation times shown in Figure 2 should yield identical relaxation rate constants for the set of experiments. If we do not know which model is the correct model, the best we can do is to parameterize the results in a way that does not depend on the model of motion, as can be done with the model-free parameters $S^{2}$ and $\langle \tau_{e}\rangle$ .

When analyzing data, a model provides a framework for understanding the data, and by using a model we are always adding some information to the experimental data. In some cases, we add further information depending on how we interpret a model. A model is advantageous if that information is correct, and disadvantageous if that information is wrong. Suppose, for example, we know that the correct model in Figure 2 is a symmetric two-site hop, shown in Figure 2C; then we may extract the hop angle and exchange rates from $S^{2}$ and $\langle \tau_{e}\rangle$ , resulting in $\theta_{\text{hop}}$ = 39° and $k_{\text{ex}}^{1\to 2}$ = $k_{\text{ex}}^{2\to 1}$ = 5 × 10^9^/s. However, if the true model is an asymmetric two-site hop, shown in Figure 2D, the true angle and exchange rates may be significantly different (for Figure 2D, these are $\theta_{\text{hop}}$ = 70° with $k_{\text{ex}}^{1\to 2}$ = 1.3 × 10^9^/s and $k_{\text{ex}}^{2\to 1}$ = 8.7 × 10^9^/s). Then, a model-free approach is the more reliable method when the correct model cannot be independently determined.

In this review, we will first discuss the original model-free approach, and then examine methods descended from it, including discussion of our own detector analysis, a relatively new approach that also provides a model-free analysis in the spirit of the original Lipari-Szabo approach, but can extract the full information content of relaxation data sets in instances where the model-free approach cannot. We discuss analysis of microsecond motions using *R*_1*ρ*_ relaxation, and finally consider how other methods, in particular molecular dynamics (MD) simulation, may be used to supply the information that NMR lacks, thus improving the interpretation of NMR parameters.

## Model-Free

While dynamics analysis methods have existed for application to solid-state NMR for some years now (Chevelkov et al., 2009b; Schanda et al., 2010; Zinkevich et al., 2013; Lamley et al., 2015a; Smith et al., 2016; Lakomek et al., 2017; Kurauskas et al., 2017), most of the approaches applied have evolved from methodology first developed for solution-state NMR. Probably the most important advance in solution-state analysis was the development of the model-free approach (Lipari and Szabo, 1982a; Lipari and Szabo, 1982b), and related two-step techniques (Wennerström et al., 1979; Halle and Wennerström, 1981; Brown, 1982). Then, we begin by reviewing some of the existing methodology, to understand advantages and disadvantages to various approaches.

### Model-Free Theory

Typical solution-state NMR data sets consist of relaxation rate constants for *R*_1_ (1/*T*_1_), *R*_2_ (1/*T*_2_), and nuclear Overhauser effect (NOE, $\sigma_{\text{IS}}$ ), acquired at one or more magnetic fields. The rate constants describe the signal decay ( $I\left(t\right)=I_{0}e^{-R_{\zeta }t}$ ) or recovery ( $I\left(t\right)=I_{\text{eq}}+\left(I_{0}-I_{\text{eq}}\right)e^{-R_{\zeta }t}$ ). In solid-state NMR, this behavior can be multi-exponential, whereas we use the rate constant that describes the powder-averaged value (Krushelnitsky et al., 2018). Relaxation is often driven by reorientation of a few anisotropic interactions, for example, for backbone ^15^N relaxation, a one-bond H–N dipole coupling and CSA are responsible for relaxation. For these experiments, the relaxation rate constants may be calculated from the spectral density, J(ω) :

$$\begin{array}{l} R_{1}^{\text{I}}=\underset{\text{dipolar}\;\text{relaxation}}{\underset{︸}{{\left(\frac{\delta^{\text{IS}}}{4}\right)}^{2}\left(J\left(\omega_{\text{I}}-\omega_{\text{S}}\right)+3J\left(\omega_{\text{I}}\right)+6J\left(\omega_{\text{I}}+\omega_{\text{S}}\right)\right)}}+\underset{\text{CSA}\;\text{relaxation}}{\underset{︸}{\frac{1}{3}{\left(\omega_{\text{I}}\Delta \sigma_{\text{I}}\right)}^{2}J\left(\omega_{\text{I}}\right)}}\\ R_{2}^{\text{I}}=\frac{1}{2}R_{1}^{\text{I}}+\underset{\text{dipolar}\;\text{relaxation}}{\underset{︸}{{\left(\frac{\delta^{\text{IS}}}{4}\right)}^{2}\left(3J\left(\omega_{\text{S}}\right)+2J\left(0\right)\right)}}+\underset{\text{CSA}\;\text{relaxation}}{\underset{︸}{\frac{2}{9}{\left(\omega_{\text{I}}\Delta \sigma_{\text{I}}\right)}^{2}J\left(0\right)}}\\ \sigma_{\text{IS}}=\underset{\text{dipolar}\;\text{relaxation}}{\underset{︸}{{\left(\frac{\delta^{\text{IS}}}{4}\right)}^{2}\left(-J\left(\omega_{\text{I}}-\omega_{\text{S}}\right)+6J\left(\omega_{\text{I}}-\omega_{\text{S}}\right)\right)}} \end{array}$$ (1)

Here, $\omega_{\text{I}}$ is the Larmor frequency (in radians/s) of the nucleus being relaxed, $\omega_{\text{S}}$ the Larmor frequency of the coupled spin (usually ^1^H), and $\delta^{\text{IS}}$ and $\Delta \sigma_{\text{I}}\omega_{\text{I}}$ are the anisotropies of the dipolar coupling and CSA, respectively ( $\delta^{\text{IS}}=-2\frac{\mu_{0}}{4\pi }\frac{h\gamma_{\text{I}}\gamma_{\text{S}}}{r_{\text{IS}}^{2}}$ , with $\mu_{0}$ the vacuum permeability in T^2^m^3^/J, $\gamma_{\text{I}},\gamma_{\text{S}}$ the gyromagnetic ratios of the two spins in radians/s, *h* is Planck’s constant in J·s, and $r_{\text{IS}}$ the distance between the spins in meters, resulting in $\delta^{\text{IS}}$ , which is the full breadth of the dipolar powder pattern in radians/s. $\Delta \sigma_{\text{I}}\omega_{\text{I}}$ is similarly the full breadth $(\Delta \sigma_{\text{I}}=\frac{3}{2}\left(\sigma_{\text{zz}}-\sigma_{\text{iso}}\right))$ of the CSA powder pattern in radians/s when the Larmor frequency of spin I is given by $\omega_{\text{I}}$ , also in radians/s (Schanda and Ernst, 2016)). The spectral density may be obtained from the Fourier transform of the correlation function of motion. The correlation function itself is the rank-2 tensor correlation function, and describes the reorientational behavior of an NMR interaction tensor in time. If we assume the correlation function is symmetric in time, we may replace $e^{i\omega t}$ with cos(ωt) in the Fourier transform. We can also change the integration bounds from (−∞,∞) to (0,∞) , and must multiply the integral by two in order to compensate for only integrating over half the space.

$$\begin{array}{l} J\left(\omega \right)=\int_{-\infty }^{\infty }C\left(t\right)e^{i\omega t}dt\\ =\int_{-\infty }^{\infty }\underset{\text{symmetric}\;\text{in}\;\text{time}}{\underset{︸}{C\left(t\right)\cos\left(\omega t\right)}}dt+i\int_{-\infty }^{\infty }\underset{\text{antisymmetric}\;\text{in}\;\text{time}\to 0}{\underset{︸}{C\left(t\right)\sin\left(\omega t\right)}}dt\\ J\left(\omega \right)=2\int_{0}^{\infty }C\left(t\right)\cos\left(\omega t\right)dt \end{array}$$ (2)

Then, model-free analysis makes a few assumptions about the correlation function:

1) The total motion of a given bond is the result of overall tumbling of the molecule in solution and internal motion of the bond within the molecule, and these two motions are statistically independent.
2) Decay of the correlation function due to internal motion is fast compared to all ω sampled by the set of experimental relaxation rate constants (i.e., the extreme narrowing limit).

The decay of the correlation due to internal motion does not need to be mono-exponential (or even multi-exponential, although we will later apply this assumption). Instead of the second assumption, we may assume that the correlation function due to internal motion is mono-exponential, in which case we do not require its decay to be fast (we will visit this case only briefly, as it is less likely to occur in practice). We also assume tumbling is isotropic, although this is not necessarily required. Note that separate methods exist in case overall tumbling and internal motion are coupled (Tugarinov et al., 2001), although we will not consider these here. As a set of equations, this yields

$$\begin{array}{l} C\left(t\right)=C^{\text{intern}\text{.}}\left(t\right)\cdot C^{\text{rot}\text{.}}\left(t\right)\\ C^{\text{rot}\text{.}}\left(t\right)=\frac{1}{5}e^{-t/\tau_{\text{M}}}\\ C^{\text{intern}\text{.}}\left(t\right)=S^{2}+\left(1-S^{2}\right)G\left(t\right)\\ G\left(0\right)=1,\;\;\;\underset{t\to \infty }{\lim}G\left(t\right)=0 \end{array}$$ (3)

The first equation is the result of statistical independence of internal and overall motion, such that we may write the total correlation function, C(t) , as a product of a correlation function resulting from the internal motion ( $C^{\text{intern}\text{.}}\left(t\right)$ ), and a correlation function resulting from the overall rotational tumbling ( $C^{\text{rot}\text{.}}\left(t\right)$ ). The overall motion may be described by a single decaying exponential, with correlation time $\tau_{\text{M}}$ if that overall motion is isotropic (occurring if the molecule is approximately spherical). For internal motion, $C^{\text{intern}\text{.}}\left(t\right)$ has an initial value of 1, and equilibrates at $S^{2}$ . $S^{2}$ is referred to as the generalized order parameter, and is related to, but not always equal to order parameters that may be extracted from measurement of residual couplings, as will be discussed in *Determining S* ^*2*^. G(t) is simply the decaying part of $C^{\text{intern}\text{.}}\left(t\right)$ , normalized such that its initial value is 1, and final value is 0. If the second assumption, fast decay of the correlation function due to internal motion is fulfilled, we may calculate J(ω) using the parameters $\tau_{\text{M}}$ , $S^{2}$ , and $\langle \tau_{e}\rangle$ , where

$$\langle \tau_{e}\rangle =\int_{0}^{\infty }e^{-t/\tau_{\text{M}}}G\left(t\right)dt$$ (4)

We calculate J(ω) in order to see how it is a function of the parameters $\tau_{\text{M}}$ , $S^{2}$ , and $\langle \tau_{e}\rangle$ .

$$\begin{array}{l} J\left(\omega \right)=\frac{2}{5}\int_{0}^{\infty }\left[S^{2}e^{-t/\tau_{\text{M}}}+\left(1-S^{2}\right)e^{-t/\tau_{\text{M}}}G\left(t\right)\right]\cos\left(\omega t\right)dt\\ =\frac{2}{5}\left[\frac{S^{2}\tau_{\text{M}}}{1+{\left(\omega \tau_{\text{M}}\right)}^{2}}+\left(1-S^{2}\right)\int_{0}^{\infty }e^{-t/\tau_{\text{M}}}G\left(t\right)\underset{\approx 1}{\underset{︸}{\cos\left(\omega t\right)}}dt\right]\\ \;=\frac{2}{5}\left[\frac{S^{2}\tau_{\text{M}}}{1+{\left(\omega \tau_{\text{M}}\right)}^{2}}+\left(1-S^{2}\right)\langle \tau_{e}\rangle \right] \end{array}$$ (5)

We see that if $e^{-t/\tau_{\text{M}}}G\left(t\right)$ decays quickly compared to ω , then we may replace cos(ωt) with 1, since the exponential approaches zero more quickly than the cosine term can evolve away from 1. Then, regardless of the precise form of G(t) , J(ω) may always be calculated from the parameters $S^{2}$ , $\langle \tau_{e}\rangle$ , and $\tau_{\text{M}}$ . Furthermore, if $\tau_{\text{M}}$ is known (usually from the analysis of *R*_1_ and *R*_2_ throughout a molecule (Kay et al., 1989)), J(ω) becomes a linear function of the parameters $S^{2}$ and $\left(1-S^{2}\right)\langle \tau_{e}\rangle$ .

Instead of assuming fast decay of G(t) , one may alternatively assume that it is mono-exponential ( $G\left(t\right)=e^{-t/\tau }$ ), yielding

$$\begin{array}{l} J\left(\omega \right)=\frac{2}{5}\int_{0}^{\infty }\left[S^{2}e^{-t/\tau_{\text{M}}}+\left(1-S^{2}\right)e^{-t/\tau_{\text{M}}}e^{-t/\tau }\right]\cos\left(\omega t\right)dt\\ {\langle \tau_{e}\rangle }^{-1}=\tau_{\text{M}}^{-1}+\tau^{-1}\\ J\left(\omega \right)=\frac{2}{5}\left[\frac{S^{2}\tau_{\text{M}}}{1+{\left(\omega \tau_{\text{M}}\right)}^{2}}+\left(1-S^{2}\right)\frac{\langle \tau_{e}\rangle }{1+{\left(\omega \langle \tau_{e}\rangle \right)}^{2}}\right] \end{array}$$ (6)

In the extreme narrowing limit, where decay of the correlation function is fast, we have $\omega \langle \tau_{e}\rangle \ll 1$ such that this result equals the result in Eq. 5. The expression in Eq. 6 is equivalent to Eq. 1 in (Lipari and Szabo, 1982a), and is valid either in the case of mono-exponential decay or fast decay of the internal correlation function. However, we find the case of fast, multi-exponential decay the more likely scenario, and so focus on this assumption.

The notation $\langle \tau_{e}\rangle$ is used to indicate the average of the effective correlation time. To understand how the integral of $e^{-t/\tau_{\text{M}}}G\left(t\right)$ is related to this average, we must assume that G(t) is the sum of decaying exponentials. This may be achieved with a sum over a discrete number of correlation times, weighted with $A_{i}$ , or a continuous distribution, defined by the function θ(z) .

$$\begin{array}{l} G\left(t\right)=\sum_{i}A_{i}e^{-t/\tau_{i}}\\ \;\;\;\text{where}\;\Sigma_{i}A_{i}=1\\ \text{–or–}\\ G\left(t\right)=\int_{-\infty }^{\infty }\theta \left(z\right)e^{-t/\left(10^{z}\cdot 1\text{ s}\right)}dz\\ \;\;\;\text{where}\;\int_{-\infty }^{\infty }\theta \left(z\right)dz=1 \end{array}$$ (7)

Since G(0)=1 , it is clear that the sum of amplitudes ( $A_{i}$ ) must be 1. For the former equation, we take a simple sum, and for the latter form, we use a distribution of correlation times, θ(z) , given on a logarithmic scale, such that $z=\log_{10}\left(\tau_{c}/\text{s}\right)$ . The distribution must similarly integrate to 1. The two forms can be treated equivalently. We have recently re-introduced the latter form (Smith et al., 2018), which was previously used to describe a variety of continuous correlation time distributions, e.g., see Beckmann (1988). We may insert this expression for G(t) (Eq. 7) into Eq. 4 in order to obtain the relationship between θ(z) and $\langle \tau_{e}\rangle$ .

$$\begin{array}{l} \langle \tau_{e}\rangle =\int_{0}^{\infty }e^{-t/\tau_{\text{M}}}\underset{G\left(t\right)}{\underset{︸}{\int_{-\infty }^{\infty }\theta \left(z\right)e^{-t/\left(10^{z}\cdot 1\text{ s}\right)}dz}}dt\\ =\int_{0}^{\infty }\int_{-\infty }^{\infty }\theta \left(z\right)e^{-t\left(\tau_{\text{M}}^{-1}+{\left(10^{z}\cdot 1\;\text{s}\right)}^{-1}\right)}dzdt\\ {\left(\tau_{e}\left(z\right)\right)}^{-1}=\tau_{\text{M}}^{-1}+{\left(10^{z}\cdot 1\;\text{s}\right)}^{-1}\\ \langle \tau_{e}\rangle =\int_{0}^{\infty }\int_{-\infty }^{\infty }\theta \left(z\right)e^{-t/\tau_{e}\left(z\right)}dzdt=\int_{-\infty }^{\infty }\theta \left(z\right){\left(-\tau_{e}\left(z\right)e^{-t/\tau_{e}\left(z\right)}\right)|}_{t=0}^{\infty }dz\\ \langle \tau_{e}\rangle =\int_{-\infty }^{\infty }\theta \left(z\right)\tau_{e}\left(z\right)dz\\ \text{equivalently}:\langle \tau_{e}\rangle =\sum_{i}A_{i}\tau_{e}^{i},\text{for}{\left(\tau_{e}^{i}\right)}^{-1}=\tau_{\text{M}}^{-1}+\tau_{i}^{-1} \end{array}$$ (8)

${\left(\tau_{e}^{i}\right)}^{-1}=\tau_{\text{M}}^{-1}+{\left(10^{z}\cdot 1\;\text{s}\right)}^{-1}$ is the effective correlation time, resulting from decay of both the correlation function due to the internal correlation time, $z=\log_{10}\left(\tau_{c}/s\right)$ and correlation time of the overall motion, $\tau_{\text{M}}$ . Since θ(z) integrates to 1, $\int_{-\infty }^{\infty }\theta \left(z\right)\tau_{e}\left(z\right)dz$ yields the weighted average of the effective correlation time, $\langle \tau_{e}\rangle$ . Then, one fits experimental data to a correlation function having the following model:

$$C^{}\left(t\right)=\frac{1}{5}(S^{2}e^{-t/\tau_{M}}+\left(1-S^{2}\right)e^{-t/\langle \tau_{e}\rangle })$$ (9)

Applying this model does not require that the true correlation function has exactly this form, but rather, the model correlation function simply must have the same values of $S^{2}$ and $\langle \tau_{e}\rangle$ as the true correlation function. In this sense, the analysis itself remains model-free, although equating $\langle \tau_{e}\rangle$ with the averaged effective correlation time requires the true correlation function to be a sum of decaying exponentials, as in Eq. 7.

### A Few Notes on Linearity

We will later note that many of the methods used for analyzing relaxation rate constants result in parameters that are linear functions of the distribution of correlation times, $\left(1-S^{2}\right)\theta \left(z\right)$ . Specifically, we mean that any parameter, $P_{m}$ , is linear to $\left(1-S^{2}\right)\theta \left(z\right)$ if it can be written as

$$P_{m}=\left(1-S^{2}\right)\int_{-\infty }^{\infty }\theta \left(z\right)p_{m}\left(z\right)dz$$ (10)

That is, for every correlation time, $z=\log_{10}\left(\tau_{c}/\text{s}\right)$ , *P* increases proportionally to $\left(1-S^{2}\right)\theta \left(z\right)$ at that correlation time, where the proportionality is defined by $p_{m}\left(z\right)$ . Furthermore, any linear combination of parameters, $P_{m}$ , is then also linear to $\left(1-S^{2}\right)\theta \left(z\right)$ , as we can see by integrating a sum of parameters, $P_{m}$ , and swapping the order of the integration and the summation.

$$\begin{array}{l} \sum_{m}a_{m}P_{m}=\sum_{m}a_{m}\left(1-S^{2}\right)\int_{-\infty }^{\infty }p_{m}\left(z\right)\theta \left(z\right)dz\\ \;\;\;\;\;\;\;\;=\left(1-S^{2}\right)\int_{-\infty }^{\infty }\underset{=\Sigma \left(z\right)}{\underset{︸}{\left[\sum_{m}a_{m}p_{m}\left(z\right)\right]}}\theta \left(z\right)dz\\ \;\;\;\;\;\;\;\;=\left(1-S^{2}\right)\int_{-\infty }^{\infty }\Sigma \left(z\right)\theta \left(z\right)dz \end{array}$$ (11)

We define the function Σ(z) to be the weighted sum of the sensitivities, $p_{m}\left(z\right)$ , which then defines the linear relationship of the sum of the $P_{m}$ to $\left(1-S^{2}\right)\theta \left(z\right)$ . This principle is one of the basic tenants of linear algebra. What can be less obvious is that a *linear fit* of parameters, $P_{m}$ , defined by a matrix, **M**, to a new set of parameters, $Q_{n}$ is also linear to $\left(1-S^{2}\right)\theta \left(z\right)$ . This is only the case if restrictions on the fit parameters, $Q_{n}$ , are not applied (no *priors* are used). In this case, the parameters $Q_{n}$ should minimize the following equation.

$$\begin{array}{l} \min\left[\sum_{m}{\left|P_{m}-{\left[M\right]}_{m,n}Q_{n}\right|}^{2}\right]\\ Q_{n}=\sum_{m}{\left[M^{-1}\right]}_{n,m}P_{m} \end{array}$$ (12)

One may determine the $Q_{n}$ by computing the pseudoinverse of **M** (denoted $M^{-1}$ ) and multiplying by the $P_{m}$ . Linearity of the $Q_{n}$ to $\left(1-S^{2}\right)\theta \left(z\right)$ results from the fact that linear combinations defined by $M^{-1}$ remain unchanged regardless of the value of the parameters being fit, $P_{m}$ . However, if the allowed values of the $Q_{n}$ are restricted with priors, then it can be that some values of $P_{m}$ will result in the latter formula in Eq. 12 yielding $Q_{n}$ outside of the allowed range. In this case, a linear least squares algorithm will search for a different solution than that given by Eq. 12, such that the $Q_{n}$ are no longer defined by $M^{-1}$ , and no longer have a consistent linear relationship to $\left(1-S^{2}\right)\theta \left(z\right)$ . Note that if priors are used, but Eq. 12 does not yield $Q_{n}$ outside of the bounds defined by the priors, then Eq. 12 still remains the best solution and linearity is maintained. In general, we will find analysis methods that rely on linear combination of data have more predictable behavior than those that do not.

Then, the model-free parameters $S^{2}$ and $\left(1-S^{2}\right)\langle \tau_{e}\rangle$ are linear to $\left(1-S^{2}\right)\theta \left(z\right)$ , because one can fit experimental relaxation rate constants with $S^{2}$ and $\left(1-S^{2}\right)\langle \tau_{e}\rangle$ (see Eq. 5), where the relaxation rate constants themselves are linear to the spectral density (Eq. 1), the spectral density is linear to the correlation function (via Fourier transform, Eq. 2), and the correlation function is linear to the distribution of correlation times, $\left(1-S^{2}\right)\theta \left(z\right)$ (Eqs. 3, 7)). Assuming the correlation function decays quickly, this linear relationship is given by the following, where $\tau_{e}\left(z\right)$ is defined in Eq. 8.

$$\begin{array}{l} S^{2}=1-\left[\left(1-S^{2}\right)\int_{-\infty }^{\infty }\theta \left(z\right)dz\right]\\ \left(1-S^{2}\right)\langle \tau_{e}\rangle =\left(1-S^{2}\right)\int_{-\infty }^{\infty }\tau_{e}\left(z\right)\theta \left(z\right)dz \end{array}$$ (13)

Note that $\langle \tau_{e}\rangle$ is not itself linear to $\left(1-S^{2}\right)\theta \left(z\right)$ , but is easily obtained from the above parameters.

### Fitting With Model-Free

**FIGURE 3:**
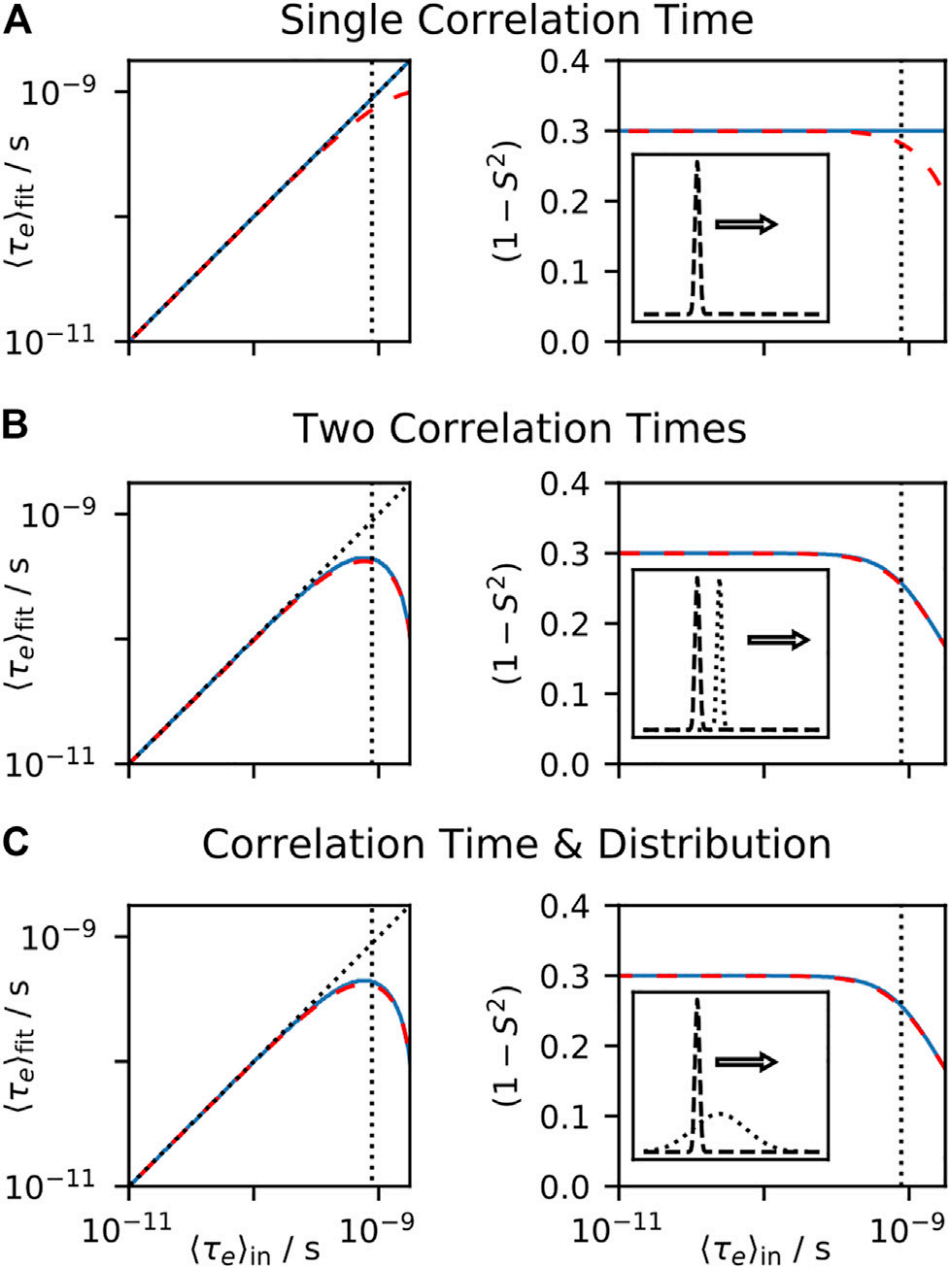
Model-free fit parameters as a function of input parameters. For each plot, a data set is calculated, using the experiments found in from Table I of Lipari and Szabo (1982b), and the resulting rate constants are fit using the model-free approach, with the resulting ${\langle \tau_{e}\rangle }_{\text{fit}}$ and $\left(1-S^{2}\right)$ shown on the left and right, respectively. For all plots, the tumbling correlation time is $\tau_{\text{M}}$ = 4 ns and $\left(1-S^{2}\right)$ = 0.3. One correlation time of the internal motion is varied, and we plot ${\langle \tau_{e}\rangle }_{\text{in}}$ on the *x*-axis. In each plot, we fit using the full spectral density (blue, solid, see Eq. 6) and using a linear approximation (red, dashed, see Eq. 5). In **(A)**, the input correlation function only has a single correlation time. In **(B)**, one correlation time is fixed to 10 ps, and the second correlation time is swept. In **(C)**, a log-Gaussian distribution (*μ* = 10 ps, *σ* = 0.75 order of magnitude) is combined with a correlation time that is varied (with total amplitude equal). On the left plots, black dotted lines indicate where the input value, ${\langle \tau_{e}\rangle }_{\text{in}}$ , matches the fit, ${\langle \tau_{e}\rangle }_{\text{fit}}$ . In all plots, vertical black dotted lines indicate where $\omega {\langle \tau_{e}\rangle }_{\text{in}}=0.5$ for ω/2π = 90 MHz, where this frequency corresponds to the highest field used for the data set.

In Figure 3, we test the performance of model-free fitting under a number of conditions. In Figure 3A, we calculate a number of relaxation rate constants from motion having a single internal correlation time and overall tumbling with $\tau_{\text{M}}$ = 4 ns, and then fit the results, assuming the model-free correlation function (Eq. 9). We may calculate the spectral density exactly, or we may assume that the correlation function decays quickly, by using the spectral density given in Eq. 5, resulting in a linear fit. The former method is shown as a blue, solid line, where the input parameters always exactly match the fit parameters, whereas using a linear fit (red, dashed line) results in disagreement of input and fit parameters when the correlation function does not decay quickly compared to the frequencies sampled ( $\omega \tau_{e}\ll 1$ ); in this case, Eq. 5 is no longer a good estimate of the spectral density whereas Eq. 6 has the correct form.

In Figure 3B, we include two correlation times in the input, each with equal amplitude, where one correlation time is fixed (10 ps), and a second correlation time is swept. We calculate the mean effective correlation time directly on the *x*-axis ( ${\langle \tau_{e}\rangle }_{\text{in}}$ ), and compare this to the fitted parameters on the *y*-axis ( ${\langle \tau_{e}\rangle }_{\text{fit}}$ , left plot, $1-S^{2}$ , right plot). As expected, if the assumption that $\omega \tau_{e}\ll 1$ holds for all frequencies sampled and all correlation times present, the fit parameters are in good agreement with their input values, but when $\omega \tau_{e}\ll 1$ , ${\langle \tau_{e}\rangle }_{\text{fit}}$ and $S^{2}$ no longer reproduce the correct values. Note that performing this fit with the full spectral density (blue, solid line) and using just a linear fit (red, dashed line) produces very similar results. In Figure 3C, we perform the same tests, but instead of fixing a correlation time to 10 ps, we have a log-Gaussian distribution of correlation times, centered at 10 ps, with a standard deviation of 0.75 orders of magnitude. Results are similar to those found in Figure 3B.

### Determining *S* ^2^

For model-free analysis, $\langle \tau_{e}\rangle$ is the average effective correlation time, and can be calculated from the distribution of correlation times. $S^{2}$ , on the other hand, is determined from the distribution of orientations sampled by internal motion. By definition, it is equal to the correlation function of internal motion, taken as the limit of *t* goes to infinity. We may obtain $S^{2}$ by first considering the formula for the correlation function.

$$C^{\text{intern}\text{.}}\left(t\right)={\langle P_{2}\left(\vec{\mu }\left(\tau \right)\cdot \vec{\mu }\left(t+\tau \right)\right)\rangle }_{\tau }$$ (14)

$P_{2}\left(x\right)$ is the second Legendre polynomial ( $P_{2}\left(x\right)=\left(3x^{2}-1\right)/2$ ), and $\vec{\mu }\left(\tau \right)$ is a normalized vector that gives the direction of the principal component of an NMR interaction as a function of time, due to internal motion only (without tumbling). The dot product ( $\vec{\mu }\left(\tau \right)\cdot \vec{\mu }\left(t+\tau \right)$ ) yields the cosine of the angle between the two vectors. The correlation function itself may take on a variety of complex forms, depending on the correlation times present, but $S^{2}$ , its value as t→∞ , depends only on the distribution of orientations sampled by the internal motion. This may be obtained by taking a weighted average over all possible starting orientations (*p*) and all possible final orientations (*q*), and calculating $P_{2}\left({\vec{\mu }}_{p}\cdot {\vec{\mu }}_{q}\right)$ for each pair. Defining $p_{\text{eq}}\left({\vec{\mu }}_{p}\right)$ to be the fraction of orientation ${\vec{\mu }}_{p}$ at thermal equilibrium, we obtain

$$S^{2}=\sum_{p}\sum_{q}p_{\text{eq}}\left({\vec{\mu }}_{p}\right)p_{\text{eq}}\left({\vec{\mu }}_{q}\right)P_{2}\left({\vec{\mu }}_{p}\cdot {\vec{\mu }}_{q}\right)$$ (15)

Then, if we have a precise description of the internal dynamics, we may calculate parameters $\langle \tau_{e}\rangle$ and $S^{2}$ using Eqs. 8, 15. We may not easily go backwards, to obtain a precise description of the dynamics from only these parameters. However, this is not a limitation of the method of analysis, but rather of the information content of the data.

In solid-state NMR, we no longer have overall tumbling motion, so the term $e^{-t/\tau_{\text{M}}}$ vanishes from the correlation function and Eq. 5 becomes simply

$$J\left(\omega \right)=\frac{2}{5}\left(1-S^{2}\right)\langle \tau \rangle$$ (16)

This prevents us from separating $S^{2}$ and 〈τ〉 via relaxation data alone (we drop the subscript *e* from τ , since it is no longer an effective correlation time); however, one may measure the size of residual couplings in NMR (Chevelkov et al., 2009a; Schanda et al., 2011), often via DIPSHIFT (Munowitz et al., 1981) or REDOR (Gullion and Schaefer, 1989). In this case, the ratio of the anisotropies of the rigid interaction ( $\delta_{\text{rigid}\text{.}}$ ) to the motionally averaged interaction ( $\delta_{\text{resid}\text{.}}$ ) defines $S_{\text{resid}\text{.}}$ .

$$S_{\text{resid}\text{.}}=\delta_{\text{resid}\text{.}}/\delta_{\text{rigid}}$$ (17)

One usually equates $S^{2}$ and $S_{\text{resid.}}^{2}$ , although for motion that does not have at least a three-fold symmetry axis, these terms are not necessarily equal (Supplementary Section S3). Examples are found in Figures 2C-E, although we see the deviation is actually quite small (e.g., $S_{\text{resid}\text{.}}^{2}$ = 0.69, vs. $S^{2}$ = 0.7), so that this approach may be used to obtain good separation of $S^{2}$ and 〈τ〉 .

## Alternative Methods

In the case that all internal motion is fast, such that the correlation function decays quickly, model-free analysis is an ideal approach for extracting dynamics information from relaxation data: the full information content of the relaxation data is captured in the parameters $S^{2}$ and $\langle \tau_{e}\rangle$ , where these parameters have simple relationships to the distribution of correlation times, $\left(1-S^{2}\right)\theta \left(z\right)$ (parameters $S^{2}$ and $\left(1-S^{2}\right)\langle \tau_{e}\rangle$ are furthermore linearly related to $\left(1-S^{2}\right)\theta \left(z\right)$ ). In case the correlation function does not decay quickly compared to the sampled frequencies, our formula for the spectral density becomes significantly more complex. To obtain it, we begin from Eq. 5 (first expression), and insert the assumed form of G(t) , found in Eq. 7, yielding the equation for the solution-state spectral density.

$$\begin{array}{l} J\left(\omega \right)=\frac{2}{5}\int_{0}^{\infty }\left[S^{2}e^{-t/\tau_{\text{M}}}+\left(1-S^{2}\right)e^{-t/\tau_{\text{M}}}\int_{-\infty }^{\infty }\theta \left(z\right)e^{-t/\left(10^{z}\cdot 1\;\text{s}\right)}dz\right]\cos\left(\omega t\right)dt\\ {\left(\tau_{e}\left(z\right)\right)}^{-1}=\tau_{\text{M}}^{-1}+{\left(10^{z}\cdot 1\;\text{s}\right)}^{-1}\\ z_{e}\left(z\right)=\log_{10}\left(\tau_{e}\left(z\right)/\text{s}\right)\\ J\left(\omega \right)=\frac{2}{5}\int_{0}^{\infty }\left[S^{2}e^{-t/\tau_{\text{M}}}+\left(1-S^{2}\right)\int_{-\infty }^{\infty }\theta \left(z\right)e^{-t/\left(10^{z_{e}(z)}\cdot 1\;\text{s}\right)}dz\right]\cos\left(\omega t\right)dt\\ =\frac{2}{5}\left[\frac{S^{2}\tau_{\text{M}}}{1+{\left(\omega \tau_{\text{M}}\right)}^{2}}+\left(1-S^{2}\right)\int_{-\infty }^{\infty }\theta \left(z\right)\frac{10^{z_{e}\left(z\right)}\cdot 1\;\text{s}}{1+{\left(\omega \cdot 10^{z_{e}\left(z\right)}\cdot 1\;\text{s}\right)}^{2}}dz\right] \end{array}$$ (18)

The first step is to combine the two exponential terms, where we define the log-effective correlation time, $z_{e}\left(z\right)$ , as a function of the log-internal correlation time, *z*, and also the rotational correlation time, $\tau_{\text{M}}$ . Subsequently, each exponential term is Fourier transformed to yield the familiar Lorentzian function. The spectral density for solid-state NMR can be similarly calculated, where the overall motion is omitted.

$$\begin{array}{l} J\left(\omega \right)=\frac{2}{5}\int_{0}^{\infty }\left(1-S^{2}\right)\int_{-\infty }^{\infty }\theta \left(z\right)e^{-t/\left(10^{z}\cdot 1\;\text{s}\right)}dz\cos\left(\omega t\right)dt\\ \;=\frac{2}{5}\left(1-S^{2}\right)\int_{-\infty }^{\infty }\theta \left(z\right)\frac{10^{z}\cdot 1\;\text{s}}{1+{\left(\omega \cdot 10^{z}\cdot 1\;\text{s}\right)}^{2}}dz \end{array}$$ (19)

The integral has a complex dependence on ω , and depends on the specific form of $\left(1-S^{2}\right)\theta \left(z\right)$ , so that by using multiple relaxation experiments, we can extract more than two parameters describing the internal motion. However, we require a different approach to extract that information. We discuss four approaches developed for treating this case: the extended model-free approach (EMF), spectral density mapping (SDM), LeMaster’s approach, and IMPACT. Another approach that bears mentioning is the slowly relaxing local structure model (SRLS), which accounts for coupling of local motional modes to overall motion of a molecule in solution (Polimeno and Freed, 1992; Tugarinov et al., 2001; Mendelman and Meirovitch, 2021; Shapiro and Meirovitch, 2012). SRLS reduces to the model-free approach as coupling between local and overall motion vanishes. However, we do not include further comparison to the analytically simpler methods discussed here.

### Extended Model-Free

Clore and coworkers found that when measuring relaxation data at higher fields (up to 600 MHz) that not all backbone motion could be well fit using the model-free approach for staphylococcal nuclease and interleukin-1β (Clore et al., 1990). They found that the simplest correlation function that could fit the data was obtained by adding another decaying exponential term, yielding the EMF correlation function.

$$C^{\text{intern}\text{.}}\left(t\right)=\left(1-S_{f}^{2}\right)e^{-t/\tau_{f}}+S_{f}^{2}\left(1-S_{s}^{2}\right)e^{-t/\tau_{s}}+S_{f}^{2}S_{s}^{2}$$ (20)

**FIGURE 4:**
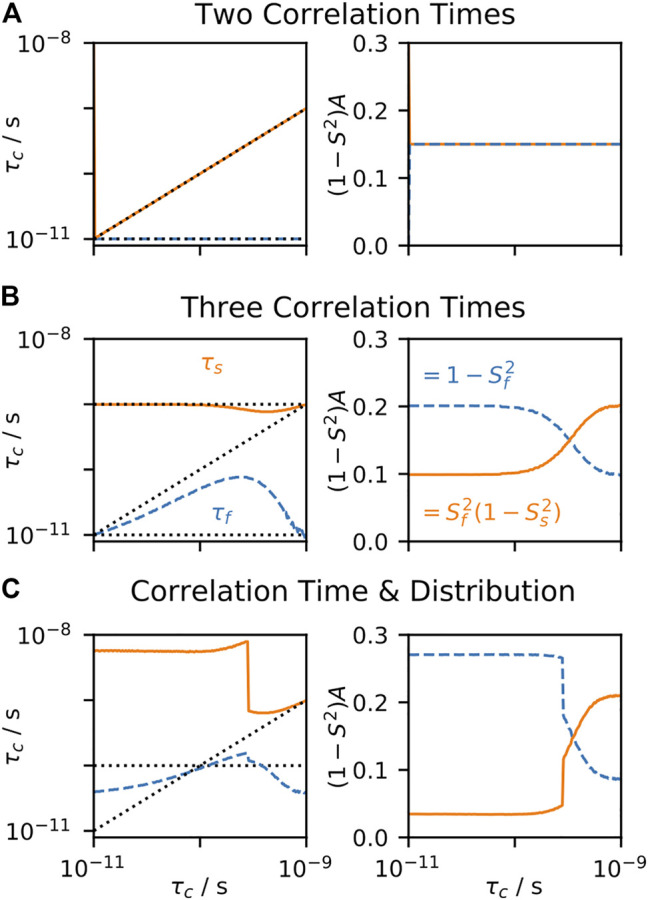
EMF parameters as a function of input correlation time (solution-state). For each plot, a data set is calculated, using the set of experiments from Clore et al. (1990), and the resulting rate constants are fitted using the EMF approach. For all plots, $\tau_{\text{M}}$ = 8.3 ns, and the input $\left(1-S^{2}\right)$ = 0.3. In each subplot, the fitted correlation times **(left)** and amplitudes **(right)** are shown, as a function of an input correlation time (*x*-axis). In **(A)**, the input correlation function has two correlation times (with equal amplitudes), with one fixed at 10 ps, and the other swept. In **(B)**, the input correlation function has three correlation times, two fixed at 10 ps and 1 ns, and the third is swept. In **(C)**, a log-Gaussian distribution of correlation times is used (*μ* = 100 ps, *σ* = 0.75 orders of magnitude), and a single correlation time is swept. Black dotted lines show the input correlation times **(left plots)**.

In this correlation function, the total internal motion is separated into fast and slow components, with order parameters $S_{f}^{2}$ and $S_{s}^{2}$ , and effective correlation times, $\tau_{f}$ and $\tau_{s}$ , respectively. The product $S_{f}^{2}S_{s}^{2}$ should yield the total order parameter, $S^{2}$ . Also note that the faster motion’s order parameter scales the influence of the slower motion, as seen in the term $S_{f}^{2}\left(1-S_{s}^{2}\right)e^{-t/\tau_{s}}$ . Data analysis with EMF in solid- and solution-state NMR involves simply varying the parameters, $S_{f}^{2}$ , $S_{s}^{2}$ , $\tau_{f}$ , and $\tau_{s}$ , to find an optimal fit to experimental data. Often, one also performs a model selection step, where one may determine how many parameters should be included in the fit (Mandel et al., 1995; d’Auvergne and Gooley, 2003; Zinkevich et al., 2013; Gill et al., 2016). In Figure 4, the behavior of EMF parameters is shown for several correlation functions. In each subplot, all terms except one correlation time are fixed, and we observe the model behavior as we sweep through the variable correlation time. In Figure 4A, two correlation times are used, so that the input correlation function has the same form as the correlation function used for fitting; as expected, the fitted parameters perfectly match the input parameters, since the input and fit models match. In Figure 4B, three correlation times are input, where the fast and slow correlation times are fixed at 10 ps and 1 ns, and the intermediate correlation time is swept. In this case, when the intermediate correlation time is fast, the fitted $\tau_{f}$ falls in between the fast and intermediate correlation times, and the fitted amplitude for the fast motion is the sum of the input amplitudes for the fast and intermediate motions. However, for longer correlation times, the fitted $\tau_{f}$ again gets shorter, eventually equaling 10 ps, so that the fitted $\tau_{s}$ takes over the role of fitting the intermediate correlation time. This is especially well illustrated in Figure 4(B, right), where the amplitude corresponding to the slow motion increases from 0.1 to 0.2, indicating that the slow motion in the model fits both the input intermediate and slow motions. Similar behavior is observed in Figure 4C, where a distribution of correlation times is combined with a single correlation time that is swept.

**FIGURE 5:**
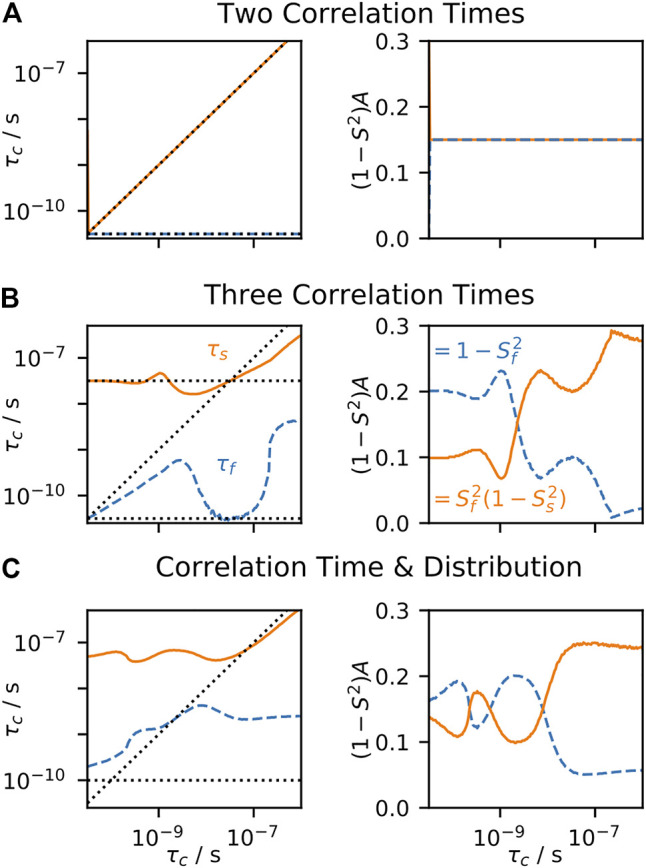
EMF parameters as a function of input correlation time (solid-state). For each plot, a data set is calculated, including direct measurement of *S*_resid._ via residual couplings (Eq. 17), ^15^N *T*_1_ at 400, 500, and 850 MHz, and *T*_2_ with MAS of 60 kHz. The resulting rate constants are fitted using the EMF approach. For all plots, $\left(1-S^{2}\right)$ = 0.3. In each subplot, the fitted correlation times **(left)** and amplitudes **(right)** are shown, as a function of an input correlation time (*x*-axis). In **(A)**, the input correlation function has two correlation times (with equal amplitudes), with one fixed at 3.2 ps, and the other swept. In **(B)**, the input correlation function has three correlation times, two fixed at 3.2 ps and 32 ns, and the third is swept. In **(C)**, a log-Gaussian distribution of correlation times is used (*μ* = 100 ps, *σ* = 0.75 orders of magnitude), and a single correlation time is swept. Black dotted lines show the input correlation times (left plots).

To the best of our knowledge, the behavior of the fit parameters has no well-defined relationship to the distribution of correlation times, $\left(1-S^{2}\right)\theta \left(z\right)$ : if we know $\left(1-S^{2}\right)\theta \left(z\right)$ precisely, our only way to obtain the EMF parameters from it would be to explicitly calculate a set of relaxation rate constants, and then fit the results to Eq. 20. This is in sharp contrast to the original model-free parameters. Similar limitations arise for the EMF approach in solid-state NMR, as seen in Figure 5. Note that typical solution-state data sets are fairly continuous in their sensitivity to motion as a function of correlation time (Smith et al., 2019a), whereas solid-state NMR has a “blind-spot” in sensitivity centered around ∼100 ns (Schanda, 2019), which results in some of the more unusual behavior for EMF in solids (see *Case 1: Extended Model-Free* for a detailed discussion of the behavior of typical model-free parameters in solid-state NMR).

### Spectral Density Mapping

In contrast to EMF, SDM is achieved by simple linear combination of sets of relaxation data at a single magnetic field (Peng and Wagner, 1992; Ishima et al., 1999). From a set of *R*_1_, *R*_2_, and NOE relaxation rate constants, one calculates

$$\begin{array}{l} J\left(0\right)=\frac{R_{2}-R_{1}/2-0.454\sigma_{\text{IS}}}{\delta_{\text{IS}}^{2}/2+2{\left(\Delta \sigma_{\text{I}}\omega_{\text{I}}\right)}^{2}}\\ J\left(\omega_{\text{I}}\right)=\frac{R_{1}-1.249\sigma_{\text{IS}}}{3{\left(\delta_{\text{IS}}/4\right)}^{2}+{\left(\Delta \sigma_{\text{I}}\omega_{\text{I}}\right)}^{2}/3}\\ J\left(0.870\omega_{\text{S}}\right)=16\sigma_{\text{IS}}/\left(5\delta_{\text{IS}}^{2}\right) \end{array}$$ (21)

The above expressions yield very close approximations of the spectral density at specific frequencies: 0, $\omega_{\text{I}}$ , and 0.870 $\omega_{\text{S}}$ , where $\omega_{\text{I}}$ is the nuclear Larmor frequency of the spin being relaxed, and $\omega_{\text{S}}$ is a spin which is dipole coupled to that spin (usually a directly bonded ^1^H). Differences in the representations of the anisotropies ( $\delta_{\text{IS}}$ , $\Delta \sigma_{\text{I}}\omega_{\text{I}}$ ) result in the different appearances of the normalization factors (denominators). These terms may be interpreted as being proportional to the amount of motion near the given frequency (which corresponds to the correlation time τ=1/ω ), but otherwise they do not provide a more physical interpretation of the motion. One may subsequently fit the spectral densities to model-free parameters for better interpretation (Gill et al., 2016). If we have a precise description of the motion (e.g., $\left(1-S^{2}\right)\theta \left(z\right)$ ), the terms J(ω) are easily obtained:

$$J\left(\omega \right)=\frac{2}{5}\left(1-S^{2}\right)\int_{-\infty }^{\infty }\theta \left(z\right)\frac{10^{z}\cdot 1\;\text{s}}{1+{\left(\omega \cdot 10^{z}\cdot 1\;\text{s}\right)}^{2}}dz\;$$ (22)

**FIGURE 6:**
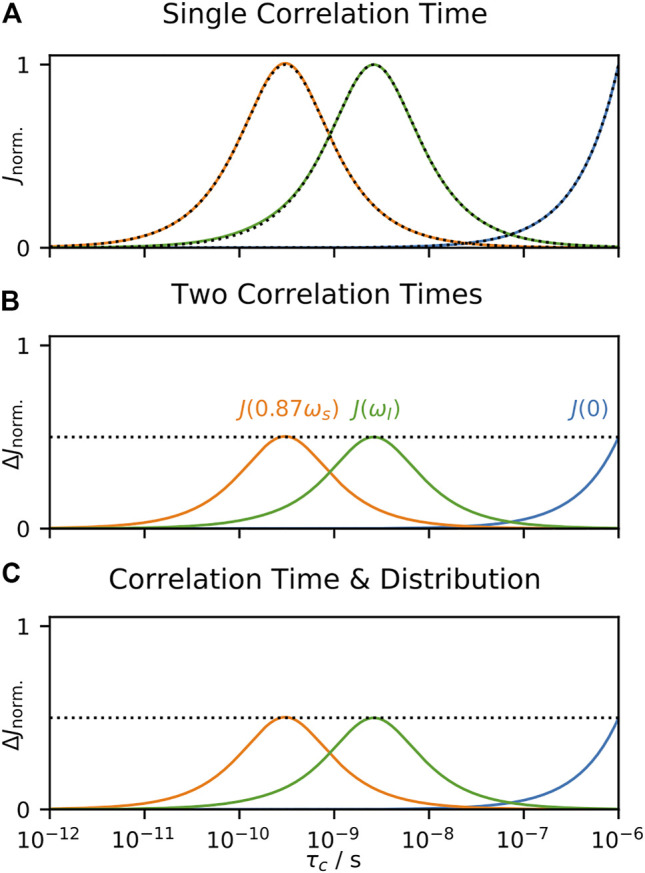
Behavior of SDM as a function of correlation time. In each subplot, we calculate ^15^N *T*_1_, *T*_2_, and σ_NH_ at 600 MHz, and analyze the results using Eq. 21. In **(A)**, the input total correlation function consists of a single decaying exponential term (with amplitude 1), where the terms J(ω) are plotted as the correlation time is varied (results are normalized). Black dotted lines show the spectral densities, J(0) , $J\left(\omega_{\text{I}}\right)$ , $J\left(0.870\omega_{\text{S}}\right)$ , calculated with Eq. 22, and colored lines show the results of the data analysis, yielding an almost exact correspondence. In **(B)**, the total correlation function now uses two correlation times (equal amplitudes), with one fixed at 10 ps, and the second swept (*x*-axis). On the *y*-axis, we plot contribution to the terms, ΔJ(ω) , from the correlation time being varied. The resulting behavior is identical to that in **(A)**, except that the amplitude is half as large, since we have split the total amplitude between the fixed and variable correlation time (dashed line marks 0.5). In **(C)**, the same information is plotted, but the total correlation function includes a log-Gaussian distribution (*μ* = 630 ps, *σ* = 1 order of magnitude), and a single, variable correlation time.

The parameters resulting from SDM always behave the same way in response to a given correlation time, regardless of other correlation times present, and is the consequence of properties of linearity discussed in *A Few Notes on Linearity*. This is seen in Figure 6A, where we calculate relaxation rate constants resulting from a single correlation time and analyze with SDM. In Figure 6B, we split motion over two correlation times, and observe how the terms respond to sweeping one of them, and in Figure 6C, we split motion into a distribution and a single, swept correlation time and determine how the terms respond to the swept correlation time. The result is always identical (scaling by 0.5 results from dividing the total amplitude into two parts), a very useful property occurring when data is analyzed strictly by linear combination of data. Unlike EMF analysis, behavior of SDM is independent of the form of the distribution of correlation times.

Note that this approach describes the total motion, and does not separate out tumbling from internal motion in the case of solution-state NMR, which has an especially strong influence on J(0) . The original approach only incorporates data from one field, whereas later work has extended the method to include data from more than one field, although one still requires specific sets of experiments (Skrynnikov et al., 2002; Hsu et al., 2018).

### LeMaster’s Approach

LeMaster proposed an alternative to SDM analysis of *R*_1_, *R*_2_, and NOE data from one field, in order to separate overall tumbling from internal motion (LeMaster, 1995). In this case, LeMaster proposed fitting data to the following correlation function:

$$\begin{array}{l} C\left(t\right)=S_{f}^{2}S_{\text{H}}^{2}S_{\text{N}}^{2}e^{-t/\tau_{\text{M}}}+S_{f}^{2}\left(1-S_{\text{H}}^{2}\right)e^{-t/\tau_{\text{H}}}+S_{f}^{2}S_{\text{H}}^{2}\left(1-S_{\text{N}}^{2}\right)e^{-t/\tau_{\text{N}}}+{\left(1-S_{f}\right)}^{2}e^{-t/\tau_{f}}\\ \tau_{\text{H}}={\left(\omega_{\text{H}}+\omega_{\text{N}}\right)}^{-1},\;\;\;\tau_{\text{N}}=|\omega_{\text{N}}{|}^{-1}\;\;\;\; \end{array}$$ (23)

It is assumed that $\tau_{f}$ is very short so that the term $\left(1-S_{f}^{2}\right)e^{-t/\tau_{f}}$ makes only negligible contributions to the spectral density, resulting in the following formula:

$$\begin{array}{l} J\left(\omega \right)=\frac{2}{5}S_{f}^{2}\left[S_{\text{H}}^{2}S_{\text{N}}^{2}\frac{\tau_{\text{M}}}{1+{\left(\omega \tau_{\text{M}}\right)}^{2}}+\left(1-S_{\text{H}}^{2}\right)\frac{\tau_{\text{H}}}{1+\left(\omega \tau_{\text{H}}^{2}\right)}+S_{\text{H}}^{2}\left(1-S_{\text{N}}^{2}\right)\frac{\tau_{\text{N}}}{1+\left(\omega \tau_{\text{N}}^{2}\right)}\right]\\ \;\;\;\;\;\;\;\;\;\;\;\;\;=\frac{2}{5}[\frac{\tau_{\text{M}}}{1+{\left(\omega \tau_{\text{M}}\right)}^{2}}+\left(1-S_{f}^{2}\right)\left(-\frac{\tau_{\text{M}}}{1+{\left(\omega \tau_{\text{M}}\right)}^{2}}\right)+\\ S_{f}^{2}\left(1-S_{\text{H}}^{2}\right)\left(\frac{\tau_{\text{H}}}{1+\left(\omega \tau_{\text{H}}^{2}\right)}-\frac{\tau_{\text{M}}}{1+{\left(\omega \tau_{\text{M}}\right)}^{2}}\right)+S_{f}^{2}S_{\text{H}}^{2}\left(1-S_{\text{N}}^{2}\right)\left(\frac{\tau_{\text{N}}}{1+\left(\omega \tau_{\text{N}}^{2}\right)}-\frac{\tau_{\text{M}}}{1+{\left(\omega \tau_{\text{M}}\right)}^{2}}\right)] \end{array}$$ (24)

**FIGURE 7:**
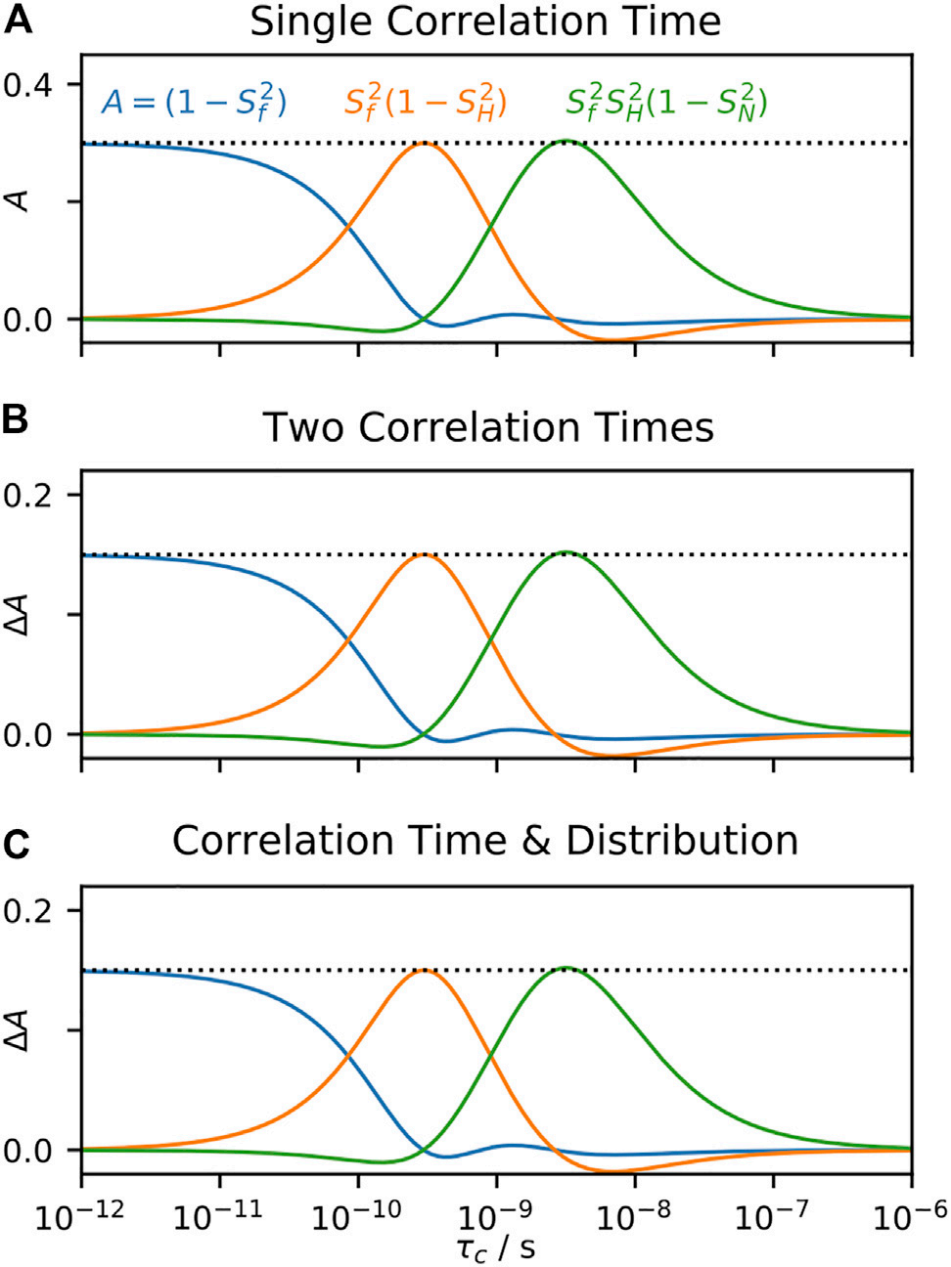
Behavior of LeMaster’s approach as a function of correlation time. In each subplot, we calculate ^15^N *T*_1_, *T*_2_, and σ_NH_ at 600 MHz for motion with $\left(1-S^{2}\right)$ = 0.3 and tumbling correlation time of $\tau_{\text{M}}$ = 4 ns, and analyze the results using Eq. 24. In **(A)** the internal correlation function consists of a single decaying exponential term (with amplitude 0.3), where the fitted amplitudes are plotted as the correlation time is varied. In **(B)** the internal correlation function uses two correlation times (both amplitudes are 0.15), with one correlation time fixed at 10 ps, and the second swept (*x*-axis). On the *y*-axis, we plot contributions to the terms from the correlation time being varied. The resulting behavior is identical to that in **(A)**, except that the amplitude is half, since we have split the total amplitude between the fixed and variable correlation time (dashed line marks 0.15). In **(C)**, the same information is plotted, but the total correlation function includes a log-Gaussian distribution (*μ* = 630 ps, *σ* = 1 order of magnitude), and a single, variable correlation time.

In the latter formulation, we find that the spectral density becomes a linear combination of terms, weighted by $\left(1-S_{f}^{2}\right)$ , $S_{f}^{2}\left(1-S_{\text{H}}^{2}\right)$ , and $S_{f}^{2}S_{\text{H}}^{2}\left(1-S_{\text{N}}^{2}\right)$ . Then, one must fit these terms to the experimental relaxation rate constants. We do so in Figure 7 for calculated relaxation rate constants. Like SDM, responses as a function of correlation time are always identical (again, excepting a scaling factor of 0.5 resulting from splitting the total motion into components), although the functions themselves are different: this results from the fact that LeMaster’s approach characterizes the internal motion, and not the total motion, so that we obtain one amplitude, $\left(1-S_{f}^{2}\right)$ , which captures information about the fastest correlation times (<30 ps), one amplitude, $S_{f}^{2}\left(1-S_{\text{H}}^{2}\right)$ , which captures information for correlation times near to $\tau_{\text{H}}$ , and one amplitude, $S_{f}^{2}S_{\text{H}}^{2}\left(1-S_{\text{N}}^{2}\right)$ , which captures information for correlation times near to $\tau_{\text{N}}$ .

LeMaster’s approach is a linear fit, without priors; as discussed in *A Few Notes on Linearity*, this means that the fitted parameters may also be obtained by a linear combination of the experimental relaxation rate constants. Therefore, the parameters $\left(1-S_{f}^{2}\right)$ , $S_{f}^{2}\left(1-S_{\text{H}}^{2}\right)$ , and $S_{f}^{2}S_{\text{H}}^{S}\left(1-S_{\text{N}}^{2}\right)$ are linear to $\left(1-S^{2}\right)\theta \left(z\right)$ . The parameters $S_{\text{H}}^{2}$ and $S_{\text{N}}^{2}$ themselves are not linear to $\left(1-S^{2}\right)\theta \left(z\right)$ , but may be obtained by simple arithmetic from the linear parameters. Like SDM, LeMaster’s approach is limited to data acquired at a single field.

### Interpretation of Motions by a Projection onto an Array of Correlation Times Approach

Limitations of the approaches above have led Ferrage and coworkers to develop the interpretation of motions by a projection onto an array of correlation times (IMPACT) approach (Khan et al., 2015), which was applied to a protein with intrinsically disordered regions (IDR). A challenge of IDRs is that the lack of structure potentially yields a large number of distinct motions and therefore many correlation times, so that EMF approach is not appropriate for data analysis, but the limited number of parameters obtained with SDM fails to provide a complete description of the dynamics. Then, the IMPACT approach allows analysis of large, multi-field data sets, by taking the total correlation function to be a sum of several fixed correlation times, $\tau_{k}$ , such that

$$C\left(t\right)=\sum_{k}A_{k}e^{-t/\tau_{k}}$$ (25)

Because C(0)=1 and decays to 0, the $A_{k}$ must sum to 1. For the Engrailed 2 protein, ^15^N *T*_1_, NOE ( $\sigma_{\text{NH}}$ ), and transverse and longitudinal cross-relaxation rate constants at five fields (400, 500, 600, 800, 1,000 MHz) could be fit to an array of six correlation times, log-spaced between 21 ps and 21 ns. When fitting to Eq. 25, one restricts the amplitudes to remain between zero and one, and the sum of amplitudes must be set to one.

**FIGURE 8:**
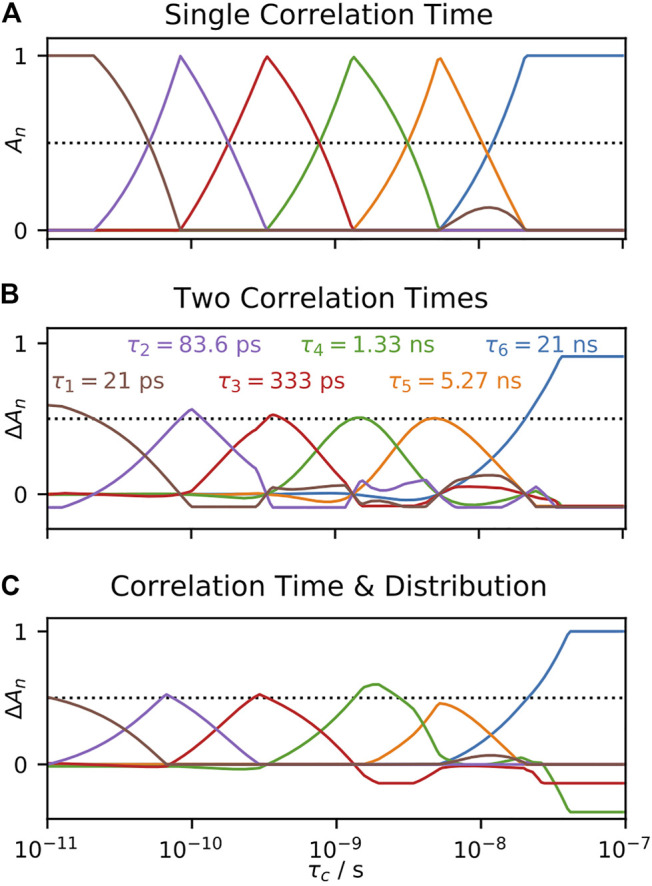
Behavior of the IMPACT approach as a function of correlation time. In each plot, we fit calculated relaxation rate constants, and fit the amplitudes in Eq. 25 according to the IMPACT procedure, using the set of experiments from Khan et al. (2015). In **(A)**, the input total correlation function consists of a single decaying exponential term (with amplitude 1), where the amplitudes are plotted as the correlation time is varied. In **(B)**, the total correlation function uses two correlation times (equal amplitudes), with one fixed at 1 ns, and the second swept (*x*-axis). On the *y*-axis, we plot contributions to the $A_{k}$ from the correlation time being varied. In **(C)**, the same information is plotted, but the total correlation function includes a log-Gaussian distribution (*μ* = 630 ps, *σ* = 1 order of magnitude), and a single, variable correlation time.

Following our procedures for SDM and LeMaster’s approach, we also examine the behavior of the IMPACT approach in Figure 8. When fitting a correlation function having a single correlation time in Figure 8A, we obtain ideal behavior from the IMPACT approach. When the input correlation time matches one of the correlation times in the IMPACT array, the corresponding amplitude is one, and all other amplitudes are zero. When the input correlation time is in between correlation times in the IMPACT array, then only the two nearest correlation times to the input value have non-zero amplitudes, and those two amplitudes sum to one (a minor deviation from this behavior occurs at 10 ns). However, if we input two correlation times in Figure 8B, or one correlation time and one distribution in Figure 8C, with motion split equally between the two correlation times or correlation time and distribution, the fit parameters’ response to the swept correlation time is not an exact reproduction of the behavior in Figure 8A, in contrast to SDM and LeMaster’s approach. While SDM and LeMaster’s approach are both linear combinations of relaxation rate constants, IMPACT is a linear fit for which its behavior depends heavily on restricting the values of the fit parameters (priors), which as discussed in *A Few Notes on Linearity*, means that the fit parameters are no longer linear to $\left(1-S^{2}\right)\theta \left(z\right)$ . The result is that the response of the parameters $A_{k}$ to a given correlation time do depend weakly on other motions present, thus not fully obtaining the ideal, linear behavior of SDM and LeMaster’s approach. However, IMPACT provides a good approximation to this behavior, and is more generally applicable than SDM and LeMaster’s approach.

**FIGURE 9:**
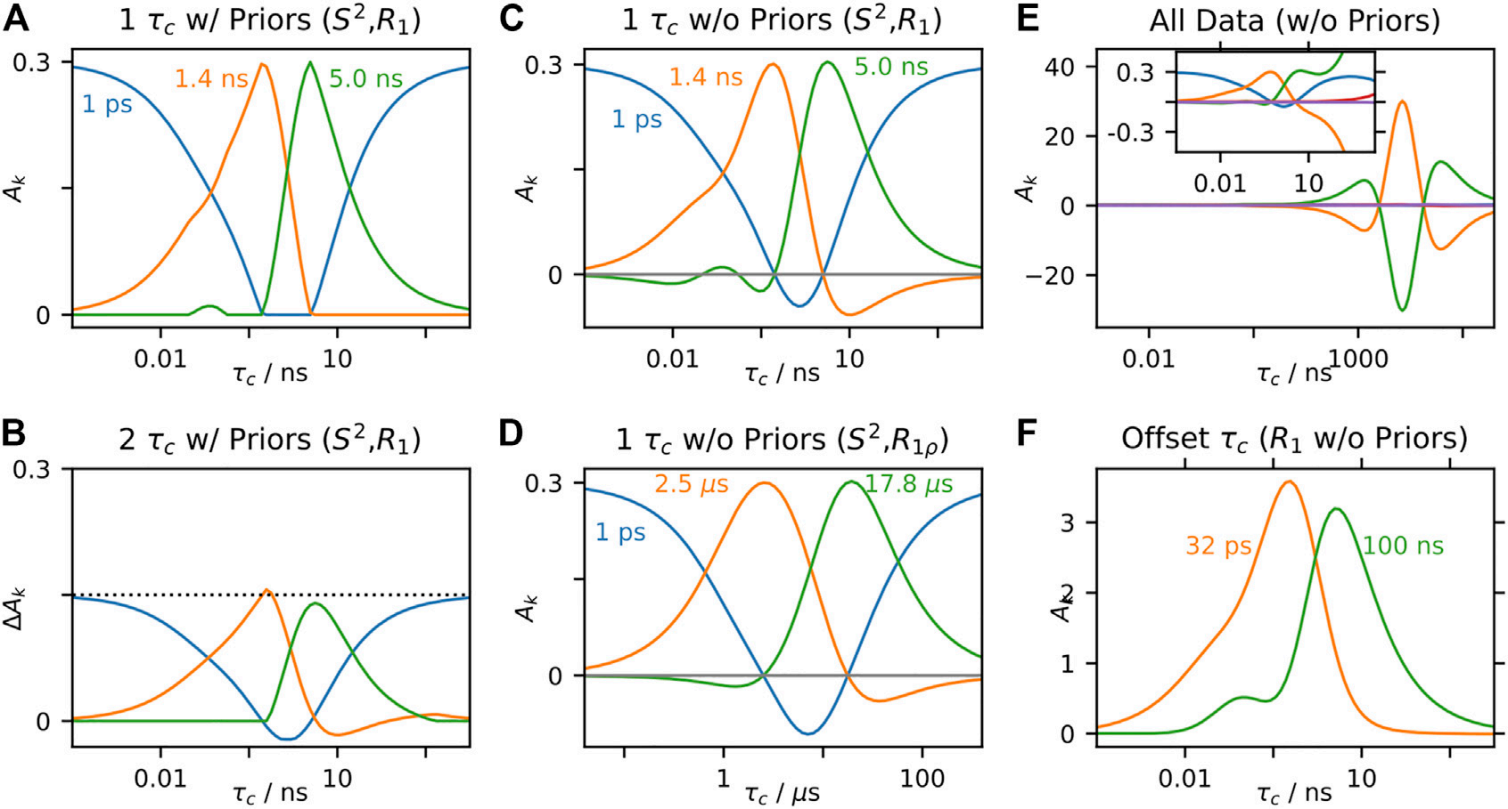
IMPACT behavior in solids. In each plot, we test the behavior of the amplitudes, $A_{k}$ , using calculated solid-state NMR data (*S* ^2^, ^15^N *R*_1_, ^15^N *R*_1*ρ*_, with experimental conditions taken from Smith et al. (2016)). **(A)** plots the behavior of fitting *S* ^2^ and three *R*_1_ rate constants to three correlation times (1 ps, 1.4 ns, 5 ns), where the input correlation function has a single correlation time ( $\left(1-S^{2}\right)$ =0.3), while restricting the $A_{k}$ to fall between 0 and 1. **(B)** shows fits under the same conditions, but includes two correlation times, with one fixed at 1 ns, and the other swept (*x*-axis). The *y*-axis plots the change in the $A_{k}$ due to the swept correlation time. **(C)** shows fits under the same conditions as **(A)**, without restricting the values of the $A_{k}$ . **(D)** also removes restrictions on the $A_{k}$ , but fits *S* ^2^ and *R*_1*ρ*_ data, using correlation times of (1 ps, 2.5 μs, and 17.8 μs). **(E)** fits all data (*S* ^2^, *R*_1_, *R*_1*ρ*_) simultaneously without restrictions on the $A_{k}$ , with correlation times of 1 ps, 1.4 ns, 5 ns, 2.5 μs, and 17.8 μs **(F)** fits *R*_1_ data, but uses one very short correlation time (32 ps), and one very long correlation time (100 ns).

IMPACT has not been developed for application to solid-state NMR, but it is worth investigating how such a method could work. In Figure 9A, we use an IMPACT-type approach to fitting *R*_1_ at three fields and *S* ^2^, using an array of three correlation times. We restrict the fitted amplitudes ( $A_{k}$ ) to fall between zero and one, but it does not make sense to require the $A_{k}$ to sum to one, since the correlation function in solid-state NMR does not usually decay to zero. Here, we assume a motion with just one correlation time, and $\left(1-S^{2}\right)$ = 0.3. Then, we find that IMPACT in solids is similar to its solution-state behavior. Note that the amplitudes corresponding to 1.4 and 5 ns capture motion near those correlation times, whereas the amplitude corresponding to 1 ps captures *all* motion not in proximity to 1.4 and 5 ns, including very slow motions. As with solution-state NMR, if we split the motion over two correlation times, and determine the response to one of the two correlation times Figure 9B, the response changes compared to fitting just the single correlation time. However, as discussed in *A Few Notes on Linearity*, and demonstrated with SDM and LeMaster’s approach, this dependence on other motions present vanishes if we eliminate restrictions on the fit parameters. Then, in Figure 9C, we repeat the fit from Figure 9A, without restrictions on the fit parameters, yielding reasonable behavior, excepting some negative amplitudes in the $A_{k}$ . Figure 9D shows similar results, when fitting *S* ^2^ and *R*_1*ρ*_, although the fitted correlation times must be in the sensitive range of the *R*_1*ρ*_ rate constants. Unfortunately, when we attempt to fit *R*_1_ and *R*_1*ρ*_ simultaneously in Figure 9E, using the same correlation times as in Figures 9C,D, we find extremely unstable behavior. Apparently, we cannot simultaneously fit data on both sides of the solid-state NMR blind spot.

In Figures 9C,D, we have fairly good performance, excepting that some of the amplitudes become slightly negative. Interestingly, these negative amplitudes may be eliminated by placing two correlation times further away from each other. Then, in Figure 9F, we fit only *R*_1_ data, using correlation times of 32 ps and 100 ns. The fitted correlation times no longer correspond to the center of the sensitive range of the $A_{k}$ (750 ps, 6.2 ns), and the amplitudes also far exceed the input value for $\left(1-S^{2}\right)$ . Fitting while also including $S^{2}$ data allows using an additional correlation time (1 ps), but the corresponding $A_{k}$ becomes large and negative (not shown). From this final result, we could simply renormalize the amplitudes to have a maximum of one, and report the center of the sensitive range instead of the correlation times to which we actually fitted. The result would still be a linear combination of the experimental data, and therefore linear to $\left(1-S^{2}\right)\theta \left(z\right)$ , but the result would have very little to do with the correlation times chosen to obtain that linear combination.

## A New Approach for Solid-State Nuclear Magnetic Resonance

In the previous section, we investigated the behavior of a number of approaches to processing relaxation data. Of those approaches, model-free, SDM, and LeMaster’s approach provide parameters which are linear to the distribution of correlation times, $\left(1-S^{2}\right)\theta \left(z\right)$ (in some cases, some additional arithmetic operations are required to obtain the reported parameters, e.g., $\langle \tau_{e}\rangle$ is calculated from $S^{2}$ and $\left(1-S^{2}\right)\langle \tau_{e}\rangle$ ). IMPACT approximates this behavior, although heavy reliance on priors prevents perfect linearity. However, each approach is limited in its application to solid-state NMR data. Therefore, we have developed the detector analysis (Smith et al., 2018), which is a general method for processing relaxation data that maintains a linear relationship between fit parameters and the distribution of correlation times.

### Linear Combination of Data

As we have emphasized for the above examples, one may obtain parameters that have a well-defined (linear) relationship to the distribution of correlation times by taking linear combinations of relaxation rate constants. Thus far, we have limited ourselves to very specific linear combinations: combinations that yield the spectral density, or combinations that are related to specific correlation times. However, why shouldn’t we use any linear combination that is optimized to give an ideal linear relationship to the distribution of correlation times, $\left(1-S^{2}\right)\theta \left(z\right)$ ? We first recall that the correlation function has been defined here as being a linear combination of decaying exponentials, defined by $\left(1-S^{2}\right)\theta \left(z\right)$ , and its Fourier transform (also a series of linear combinations) must then also be linear to $\left(1-S^{2}\right)\theta \left(z\right)$ .

$$\begin{array}{l} C\left(t\right)=S^{2}+\left(1-S^{2}\right)\int_{-\infty }^{\infty }\theta \left(z\right)e^{-t/\left(10^{z}\cdot 1\;\text{s}\right)}dz\\ J^{\left(\theta ,S\right)}\left(\omega \right)=\frac{2}{5}\left(1-S^{2}\right)\int_{-\infty }^{\infty }\theta \left(z\right)\frac{10^{z}\cdot 1\;\text{s}}{1+{\left(\omega \cdot 10^{z}\cdot 1\;\text{s}\right)}^{2}}dz \end{array}$$ (26)

Here, we take $J^{\left(\theta ,S\right)}\left(\omega \right)$ to be the spectral density resulting from $\left(1-S^{2}\right)\theta \left(z\right)$ . Then, any relaxation rate constant is a weighted sum of terms from the spectral density.

$$\begin{array}{l} R_{\zeta }^{\left(\theta ,S\right)}=\sum_{p}a_{p}^{\zeta }J^{\left(\theta ,S\right)}\left(\omega_{p}\right)\\ \;\;\;\;\;\;\;=\sum_{p}a_{p}^{\zeta }\left(1-S^{2}\right)\int_{-\infty }^{\infty }\theta \left(z\right)\frac{10^{z}\cdot 1\;\text{s}}{1+{\left(\omega \cdot 10^{z}\cdot 1\;\text{s}\right)}^{2}}dz\\ =\left(1-S^{2}\right)\int_{-\infty }^{\infty }\theta \left(z\right)\underset{=R_{\zeta }\left(z\right)}{\underset{︸}{\sum_{p}a_{p}\frac{10^{z}\cdot 1\;\text{s}}{1+{\left(\omega \cdot 10^{z}\cdot 1\;\text{s}\right)}^{2}}}}dz\\ R_{\zeta }^{\left(\theta ,S\right)}=\left(1-S^{2}\right)\int_{-\infty }^{\infty }\theta \left(z\right)R_{\zeta }\left(z\right)dz \end{array}$$ (27)

$R_{\zeta }^{\left(\theta ,S\right)}$ is the relaxation rate constant for an experiment, indexed *ζ,* resulting from the distribution of correlation times, $\left(1-S^{2}\right)\theta \left(z\right)$ . Coefficients $a_{p}^{\zeta }$ indicate the weightings of the spectral density for experiment *ζ*, sampled at frequencies $\omega_{p}$ . Insertion of $J^{\left(\theta ,S\right)}\left(\omega \right)$ into this linear combination allows us to express $R_{\zeta }^{\left(\theta ,S\right)}$ as a linear function of $\left(1-S^{2}\right)\theta \left(z\right)$ , where $R_{\zeta }\left(z\right)$ defines the linear relationship (we refer to this as the sensitivity).

Then, as is the case for model-free, SDM, and LeMaster’s approach, any sum of relaxation constants maintains linearity. Following our previous convention (Smith et al., 2018), we denote the sum as $\rho_{n}^{\left(\theta ,S\right)}$ .

$$\begin{array}{l} \rho_{n}^{\left(\theta ,S\right)}=\sum_{\zeta }b_{\zeta }R_{\zeta }^{\left(\theta ,S\right)}\\ \;\;\;\;\;\;\;\;\;\;\;=\sum_{\zeta }b_{\zeta }\left(1-S^{2}\right)\int_{-\infty }^{\infty }\theta \left(z\right)R_{\zeta }\left(z\right)dz\\ =\left(1-S^{2}\right)\int_{-\infty }^{\infty }\theta \left(z\right)\underset{=\rho_{n}\left(z\right)}{\underset{︸}{\sum_{\zeta }b_{\zeta }R_{\zeta }\left(z\right)}}dz\\ \rho_{n}^{\left(\theta ,S\right)}=\left(1-S^{2}\right)\int_{-\infty }^{\infty }\theta \left(z\right)\rho_{n}\left(z\right)dz \end{array}$$ (28)

Then, $\rho_{n}\left(z\right)$ defines the linear relationship between $\left(1-S^{2}\right)\theta \left(z\right)$ and $\rho_{n}^{\left(\theta ,S\right)}$ . The subsequent question is, how do we find the best linear combinations of the experimental relaxation rate constants for analyzing our relaxation data?

### Optimizing Detectors: The Relaxation-Rate Space Approach

While any linear combination of experimental relaxation rate constants yields a linear relationship between $\left(1-S^{2}\right)\theta \left(z\right)$ and the resulting $\rho_{n}^{\left(\theta ,S\right)}$ , not all combinations are equally good choices. A few guidelines are, first, non-negativity of $\rho_{n}\left(z\right)$ ; we would like $\rho_{n}^{\left(\theta ,S\right)}$ to always increase when amplitude of motion increases, whereas negative regions of $\rho_{n}\left(z\right)$ could cause $\rho_{n}^{\left(\theta ,S\right)}$ to decrease with increasing amplitudes. Second, narrowness: we would like each $\rho_{n}^{\left(\theta ,S\right)}$ to report on a specific range of correlation times. Third, when the full set of relaxation data is analyzed, one should be able to back-calculate the experimental data (within some tolerance) from the parameters $\rho_{n}^{\left(\theta ,S\right)}$ . This ensures that one captures all information in the experimental data (clearly, if the $\rho_{n}^{\left(\theta ,S\right)}$ can reproduce the experimental data, then the $\rho_{n}^{\left(\theta ,S\right)}$ must have retained the information in the experiments).

The question, then, is how to obtain optimized linear combinations satisfying the above requirements. Our initial answer to this question is the result of identifying a similar problem in a completely different field: When one sees the color of an object, its appearance depends on the distribution of wavelengths reflected (or emitted) by the object. The distribution of wavelengths is given by the spectral power distribution, S(λ) . Whereas S(λ) is an infinite-dimensional description of the spectral power vs. wavelength, what is “seen” is a projection of that distribution onto a three dimensional space, corresponding to the three cones that detect color in the eye. This 3D space is often described using the CIE (Commission internationale de l'Eclairage) XYZ color space (Smith and Guild, 1931; Judd, 1951; Vos, 1978).

$$\begin{array}{l} X=\int_{0}^{\infty }S\left(\lambda \right)\bar{x}\left(\lambda \right)d\lambda \\ Y=\int_{0}^{\infty }S\left(\lambda \right)\bar{y}\left(\lambda \right)d\lambda \\ Z=\int_{0}^{\infty }S\left(\lambda \right)\bar{z}\left(\lambda \right)d\lambda \end{array}$$ (29)

**FIGURE 10:**
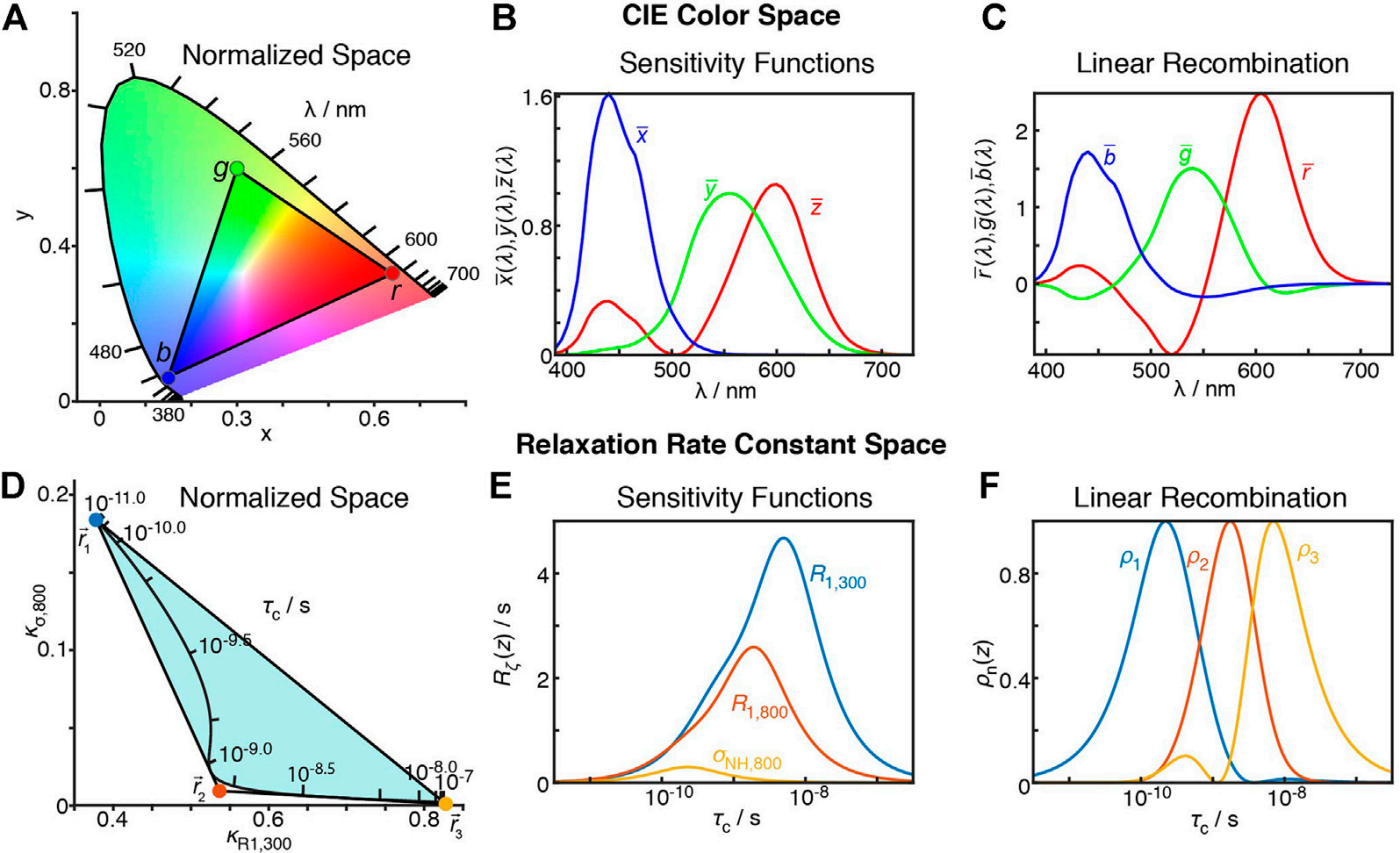
Similarity between the CIE XYZ colorspace and the relaxation rate constant space. **(A)** plots the XYZ colorspace, black lines indicate where single wavelengths fall in the colorspace (*z* not shown, space is normalized such that *x* + *y* + *z* = 1). Points connected by a triangle indicate the definition of red, green, and blue colors as defined by the sRGB standard (Anderson et al., 1996). **(B)** plots the sensitivity of the $\bar{x}\left(\lambda \right)$ , $\bar{y}\left(\lambda \right)$ , and $\bar{z}\left(\lambda \right)$ color matching functions as a function of wavelength (*λ*). **(C)** plots sRGB sensitivities resulting from transformation from the XYZ to sRGB spaces. Points connected by triangles correspond to definitions of ${\vec{r}}_{1}$ , ${\vec{r}}_{2}$ , and ${\vec{r}}_{3}$ that define the detector space. **(D)** shows the normalized relaxation rate space for ^13^C *R*_1_ at 300 and 800 MHz and H–C NOE at 800 MHz. **(E)** shows the sensitivities of each of these experiments a function of correlation time. **(F)** shows detector sensitivities resulting from transformation from the relaxation rate constant space to detector space (defined by the points in **(D)**).

The functions $\bar{x}\left(\lambda \right)$ , $\bar{y}\left(\lambda \right)$ , and $\bar{z}\left(\lambda \right)$ are plotted in Figure 10B. Based on the color one sees, one cannot define S(λ) precisely, but certainly we learn something about the distribution of wavelengths. In the same way, based on a set of relaxation rate constants, we cannot fully define $\left(1-S^{2}\right)\theta \left(z\right)$ , but certainly we can learn something about the dynamics. The matching forms of Eqs. 27, 29 further highlight the relationship between these problems.

The XYZ color space can be represented as a 2D space, shown in Figure 10A. Only *x* and *y* are shown, and *z* is selected so that x+y+z=1 (then, a third dimension would vary this sum, corresponding to brightness). By marking points in the color space, one can indicate how the color space may be represented in another basis. Here, we have marked points corresponding to red, green, and blue of the sRGB standard (Anderson et al., 1996). Colors within the resulting triangle may be obtained with positive linear combinations of the red, green, and blue of sRGB, so that this triangle is a good estimate of colors that may be obtained with a color monitor (which creates color by combining red, green and blue pixels–this means that in Figure 10A, colors outside the triangle are not correctly represented on your screen). These points also define a transformation from the XYZ color matching functions (Figure 10B) to the sRGB functions (Figure 10C). Note that any color may be represented in the sRGB space, but only those where S(λ) results in positive R, G, and B values can actually be reproduced by a typical monitor.

Realizing that the mathematics of relaxation rate constants was essentially equivalent to color spaces, we created analogous relaxation rate constant spaces, replacing the *X*, *Y*, and *Z* values with normalized rate constants. However, instead of placing points within the relaxation rate space, we surrounded the space in Figure 10D, since we wanted to describe all points in the space with *positive* parameters. Interestingly, by surrounding the space as closely as possible, without crossing into the space, we obtained a transformation to functions with well-separated and non-negative sensitivities, see Figure 10F. In the example here, we use three points to transform the three experimental sensitivities into detector sensitivities, resulting in three detectors. However, redundancy in the information of larger data sets often results in the space becoming narrow in a given dimension, so that the full space may also be approximately described using fewer points, resulting in fewer detectors than experimental data points, but better signal-to-noise in the resulting parameters. Full details of this approach are described in Smith et al. (2018).

### Optimizing Detector Sensitivities: Automated Approach

Investigating the relaxation rate space is a powerful way to grasp the information content of a relaxation data set, however, detector optimization using this method requires manual selection of points in the space. This quickly became excessively tedious for large data sets, as is the case for analysis of relaxometry data (Smith A. A. et al., 2021), so that we have also automated the optimization of linear combination (Smith et al., 2019a).

For automation, one still has the requirements that we capture the information in the experiments (that is, we can fit the data), while minimizing the number of parameters to describe that data, and second, that we obtain detector sensitivities that are narrow and non-negative. The first requirement may be met using singular value decomposition (Golub and Kahan, 1965). Suppose we have a matrix, **M**, for which each row is a sensitivity of one of our experiments ( $R_{\zeta }\left(z\right)$ ), where we perform a normalization to prioritize fitting of higher quality data (procedure: first, we normalize all sensitivities to a maximum of one, second we multiply the sensitivity by the median of the experimental rate constants, and third we divide by the median standard deviation of those rate constants). Each column then corresponds to a correlation time. For *N* experiments, we obtain the best approximation of **M** that can be achieved with a linear combination of *t* vectors, defined by

$$\begin{array}{l} M\approx \tilde{M}=U_{t}\cdot \Sigma_{t}\cdot V_{t}^{\prime }\\ V_{t}^{\prime }=\Sigma_{t}^{-1}\cdot U_{t}^{\prime }\cdot M \end{array}$$ (30)

The *t* rows of $V_{t}^{\prime }$ are linear combinations of the rows of **M**, with recombination defined by the product $\Sigma_{t}^{-1}\cdot U_{t}^{\prime }\cdot M$ ( $U_{t}$ , $V_{t}^{\prime }$ are unitary matrices, and $\Sigma_{t}$ is diagonal, with the largest *n* singular values along the diagonal). Linear combination of the rows of $V_{t}^{\prime }$ to yield the rows of **M** is an approximate relationship, but the inverse, recombination of the rows of **M** to yield $V_{t}^{\prime }$ , is exact. Then, the closer $\tilde{M}$ is to **M**, the better the data can be fit, but this requires *t* to be larger, and thus more noise is also present in the final analysis. In principle, this linear recombination could be directly applied to the experimental data, to obtain detectors with sensitivities given by the rows of the $V_{t}^{\prime }$ . The result would capture (approximately) the maximum amount of information possible from the experiment with *t* parameters. However, the sensitivities found in the rows of $V_{t}^{\prime }$ are not narrow, and usually have large negative regions. On the other hand, a linear recombination of the vectors in $V_{t}^{\prime }$ would maintain the information content and fit quality, but allows one to optimize the detector sensitivities to be separated and non-negative.

$$\left[\begin{array}{c} \rho_{1}\left(z\right)\\ \rho_{2}\left(z\right)\\ \vdots \end{array}\right]=T\cdot V_{t}^{\prime }=T\cdot \Sigma_{t}^{-1}\cdot U_{t}^{\prime }\cdot M$$ (31)

Then, **T** defines the linear recombination of the $V_{t}^{\prime }$ to yield the $\rho_{n}\left(z\right)$ , where **T** is a square matrix. The product of a row of **T** with $V_{t}^{\prime }$ defines one of the detectors sensitivities, $\rho_{n}\left(z\right)$ . A row of **T** is determined in order to optimize a detector sensitivity, first by choosing a single correlation time, $z_{\text{max}}=\log_{10}\left(\tau_{c}/\text{s}\right)$ , for which we optimize a linear combination of the rows of $V_{t}^{\prime }$ such that $\rho_{n}\left(z_{\text{max}}\right)$ = 1, while simultaneously minimizing $\rho_{n}\left(z\right)$ for all other correlation times, and requiring that all $\rho_{n}\left(z\right)$ remain non-negative. This can be quickly solved using a linear programming algorithm (Kantorovich, 1960; Dantzig, 1982; Virtanen, 2020). However, if we sweep through an array of correlation times, performing this optimization at each correlation time, we find that we are only successful at *t* correlation times (we consider the minimization as having failed if for some *z*, we find that $\rho_{n}\left(z\right)$ exceeds 1). Currently, we find the best *t* detectors by sweeping over a large array of correlation times (200), although this algorithm could be improved to reduce the number of optimizations required (spaces method and automated method both implemented in MATLAB, download from https://difrate.sourceforge.io).

In the detector analysis, once we have optimized the detectors, we apply the same linear combination to the experimental relaxation rate constants as were applied to the sensitivities in order to obtain optimized detector responses. Note in practice that this is implemented as a fit, allowing one to prioritize fitting relaxation rate constants with lower measurement error. Furthermore, we place bounds on the fitted detector responses, $\rho_{n}^{\left(\theta ,S\right)}$ . In *A Few Notes on Linearity*, we noted that bounds (priors) on the fit parameters can cause the fit parameters to not be linear to $\left(1-S^{2}\right)\theta \left(z\right)$ . This is only the case if the priors exclude the best fit. Detectors are constructed such that any *allowed* set of relaxation rate constants will not result in parameters that violate the priors. Allowed rate constants are any set that may occur for an arbitrary form of $\left(1-S^{2}\right)\theta \left(z\right)$ . If, due to noise or measurement error, a dis-allowed set of relaxation rate constants is measured, then the priors will force the fitted relaxation rate constants to fall in the allowed space.

## Model-Free, or Not?

We see that the original model-free approach, SDM, LeMaster’s approach, and detector analysis all belong to a family of methods that yield parameters with well-defined relationships to the distribution of correlation times, here defined by $\left(1-S^{2}\right)\theta \left(z\right)$ . For SDM and detectors, the final parameters ( J(ω) , $\rho_{n}^{\left(\theta ,S\right)}$ ) are linearly related to $\left(1-S^{2}\right)\theta \left(z\right)$ ; for model-free, $S^{2}$ and $\left(1-S^{2}\right)\langle \tau_{e}\rangle$ are linear, and for LeMaster’s approach, $\left(1-S_{f}^{2}\right)$ , $S_{f}^{2}\left(1-S_{\text{H}}^{2}\right)$ , and $S_{f}^{2}S_{\text{H}}^{2}\left(1-S_{\text{N}}^{2}\right)$ are linear, whereas the final parameters ( $S^{2}$ , $\langle \tau_{e}\rangle$ , $S_{f}^{2}$ , $S_{\text{H}}^{2}$ , and $S_{\text{N}}^{2}$ ) must be obtained via additional arithmetic operations. Response of EMF parameters, on the other hand, may react to changes in one motion differently, depending on other motions in the system. Still, its simplicity in analysis and interpretation—one to three pairs of correlation times and amplitudes—makes it an attractive choice for relaxation data analysis. Should we then compromise in some cases, and sacrifice well-defined parameters for more easily interpreted parameters?

### Case 1: Extended Model-Free

**FIGURE 11:**
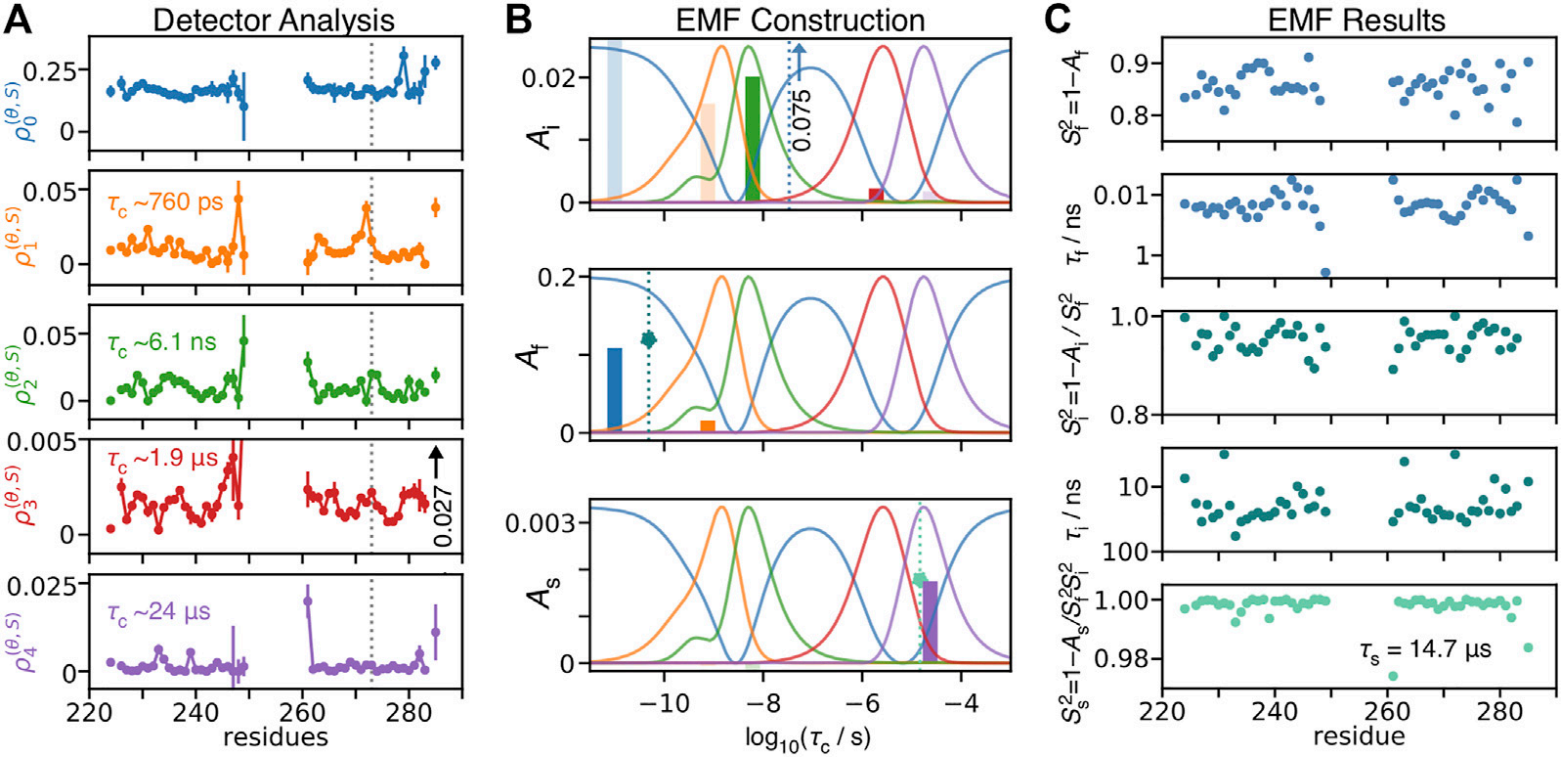
Model-free analysis from detectors. **(A)** shows a detector analysis of HET-s (218–289) fibrils (Smith et al., 2016), with sensitivities shown in **(B)** (amplitude scale not shown; sensitivities have a maximum of 1). **(B)** illustrates the procedure to convert 273Ser detector responses into model-free parameters. Bars give the detector responses (*y*-axis), plotted at the center of the corresponding detector’s sensitivity (*x*-axis, note that *ρ*_0_, blue, does not have a well-defined center). At top, we find the ratio of $\rho_{3}^{\left(\theta ,S\right)}/\rho_{2}^{\left(\theta ,S\right)}$ is consistent with a correlation time of 34 ns, with corresponding amplitude of 0.075 (intermediate motion). After subtracting the contribution of this correlation time to $\rho_{0}^{\left(\theta ,S\right)}$ (middle), we find the ratio $\rho_{1}^{\left(\theta ,S\right)}/\rho_{0}^{\left(\theta ,S\right)}$ is consistent with a correlation time of 49 ps, and amplitude of 0.12 (fast motion). Using a fixed correlation time of 14.7 μs, we find an amplitude for the slow motion of 1.8 × 10^−3^ (bottom). **(C)** shows the results of EMF analysis for all residues using the procedure in **(B)**.

Using detectors, we may better understand how EMF parameters in solid-state NMR depend on amplitudes of motion for particular windows of correlation times. We re-analyze relaxation data of HET-s (218–289) fibrils (Smith et al., 2016), by first performing a detector analysis on the data, shown in Figure 11A and then iteratively fitting detector responses to correlation times and amplitudes in Figure 11B, resulting in the EMF analysis in Figure 11C.

Using the following procedure, we are able to reproduce our previous model-free results, illustrated in Figure 11B for residue 273Ser. The procedure is given below as a set of simple equations, where results are a good reproduction of our previous direct fit using the model-free approach.

$$\begin{array}{l} \begin{array}{lll} \begin{array}{l} \mathrm{Step}\;1:z_{\text{i}}\\ \frac{\rho_{2}^{\left(\theta ,S\right)}}{\rho_{3}^{\left(\theta ,S\right)}}=\frac{\rho_{2}\left(z_{\text{i}}\right)}{\rho_{3}\left(z_{\text{i}}\right)} \end{array} & \; & \begin{array}{l} \mathrm{Step}\;2:A_{\text{i}}\\ A_{\text{i}}=\frac{\rho_{2}^{\left(\theta ,S\right)}}{\rho_{2}\left(z_{\text{i}}\right)}=\frac{\rho_{3}^{\left(\theta ,S\right)}}{\rho_{3}\left(z_{\text{i}}\right)} \end{array}\\ \begin{array}{l} \mathrm{Step}\;3:z_{\text{i}}\\ \frac{\rho_{0}^{\left(\theta ,S\right)}-A_{\text{i}}\rho_{0}\left(z_{\text{i}}\right)}{\rho_{1}^{\left(\theta ,S\right)}-A_{\text{i}}\rho_{1}\left(z_{\text{i}}\right)}=\frac{\rho_{0}\left(z_{\text{f}}\right)}{\rho_{1}\left(z_{\text{f}}\right)} \end{array} & \; & \begin{array}{l} \mathrm{Step}\;4:A_{\text{f}}\\ A_{\text{f}}=\frac{\rho_{0}^{\left(\theta ,S\right)}-A_{\text{i}}\rho_{0}\left(z_{\text{i}}\right)}{\rho_{0}\left(z_{\text{f}}\right)}=\frac{\rho_{1}^{\left(\theta ,S\right)}-A_{\text{i}}\rho_{1}\left(z_{\text{i}}\right)}{\rho_{1}\left(z_{\text{f}}\right)} \end{array} \end{array}\\ \mathrm{Step}\;5:A_{\text{s}}\\ A_{\text{f}}=\frac{\rho_{4}^{\left(\theta ,S\right)}-A_{\text{i}}\rho_{4}\left(z_{\text{i}}\right)-A_{\text{f}}\rho_{4}\left(z_{\text{f}}\right)}{\rho_{4}\left(z_{\text{s}}\right)}\\ \begin{array}{ccc} \begin{array}{l} S_{\text{f}}^{2}=1-A_{\text{f}}\\ S_{\text{i}}^{2}=1-A_{\text{i}}/S_{\text{f}}^{2}\\ S_{\text{s}}^{2}=1-A_{\text{s}}/\left(S_{\text{f}}^{2}S_{\text{i}}^{2}\right) \end{array} & \; & \begin{array}{l} \tau_{\text{f}}=10^{z_{\text{f}}}\cdot 1\text{ s}\\ \tau_{\text{i}}=10^{z_{\text{i}}}\cdot 1\text{ s}\\ \tau_{\text{s}}=10^{z_{\text{s}}}\cdot 1\text{ s} \end{array} \end{array} \end{array}$$ (32)

In the first and second steps, we find a correlation time for which the ratio of sensitivities of *ρ*_2_ and *ρ*_3_ matches the ratio of the detector responses, and then subsequently find the correct amplitude to reproduce these correlation times. With $\rho_{2}^{\left(\theta ,S\right)}$ = 2.0 × 10^−2^, and $\rho_{3}^{\left(\theta ,S\right)}$ = 2.2 × 10^−3^, we find $\tau_{\text{i}}$ = 34 ns. Our first concern with this fit is that the intermediate correlation time, $z_{\text{i}}=\log_{10}\left(\tau_{\text{i}}/\text{s}\right)$ , is a compromise between a detector sensitive to motions around 6 ns and a second sensitive around 2 μs. It seems unlikely that the same motion can really explain these two detector responses, which have sensitivities separated by three orders of magnitude. The second problem is because we use a compromise correlation time, both detector sensitivities are very low at this correlation time, which must be counterbalanced by using a large amplitude ( $A_{\text{i}}$ ) in the model-free fit. Then, in our example, $A_{\text{i}}$ = 0.075 is significantly larger than the detector responses, $\rho_{2}^{\left(\theta ,S\right)}$ and $\rho_{3}^{\left(\theta ,S\right)}$ , from which it results, so that we are very likely overestimating the amplitude of this motion.

In the third and fourth steps, we subtract the contributions from $z_{\text{i}}$ and $A_{\text{i}}$ from $\rho_{0}^{\left(\theta ,S\right)}$ and $\rho_{1}^{\left(\theta ,S\right)}$ , and similarly use the ratios of the remainder of the detector responses to obtain $z_{\text{f}}$ , and their amplitudes to obtain $A_{\text{f}}$ . Again, it is not clear if these detectors should be treated as if they describe a single motion. In particular, the relatively uniform behavior of $\rho_{0}^{\left(\theta ,S\right)}$ likely is a result of primarily local librational motion, which will not be described by the same amplitudes and correlation times of motions leading to greater variation in $\rho_{1}^{\left(\theta ,S\right)}$ . Interestingly, because the amplitudes do not vary in the same way, the variation in amplitude of $\rho_{1}^{\left(\theta ,S\right)}$ cannot be reproduced in the trends for $A_{\text{f}}$ , but instead has to be fitted by variation in correlation time ( $\tau_{\text{f}}$ ). The result is that amplitude trends in $S_{\text{i}}^{2}=1-A_{\text{i}}/S_{\text{f}}^{2}$ , shown in Figure 11(C, middle) correlate well with trends in $\tau_{\text{f}}$ , especially near breaks between the β-sheets of HET-s (near 235Glu, 271Gly). However, this correlation is actually coming from similar amplitude trends observed for $\rho_{1}^{\left(\theta ,S\right)}$ and $\rho_{2}^{\left(\theta ,S\right)}$ . The corresponding detector sensitivities are centered at 760 ps and 6.1 ns, and in fact overlap, suggesting that they may describe the same or at least related motions. EMF attributes these detector responses to different motions, having median correlation times of 22 ps and 42 ns (taken over all residues), thus being separated by three orders of magnitude.

In the final step, one fixes the slow correlation time to 14.7 μs (based on a fit optimization over the whole data set). In this case, the amplitude of $\rho_{4}^{\left(\theta ,S\right)}$ determines $A_{\text{s}}$ alone; the proximity of 14.7 μs to the center of $\rho_{4}$ (24 μs) results in fairly reasonable amplitudes (for 273Ser, $\rho_{4}^{\left(\theta ,S\right)}$ and $A_{\text{s}}$ fall within rounding error, yielding 1.8 × 10^−3^).

Then, the major problems with this EMF analysis are intermediate correlation times falling within the NMR blind spot (∼20–600 ns), along with correspondingly inflated amplitudes, as well as similar problems due to fitting fast correlation times to $\rho_{0}^{\left(\theta ,S\right)}$ and $\rho_{1}^{\left(\theta ,S\right)}$ , which requires a compromise correlation time between librational motions (∼ps) with nanosecond motions. Furthermore, this behavior prevented comparison of EMF parameters for HET-s to MD results, whereas detectors yielded reasonable agreement (Smith et al., 2019b). As we have previously pointed out (Smith et al., 2017), the model-free parameters in HET-s fibrils are far from being atypical, in fact they are fairly consistent across multiple protein systems, likely due most studies utilizing similar data sets and analysis methodology (Chevelkov et al., 2009b; Schanda et al., 2010; Haller and Schanda, 2013; Zinkevich et al., 2013; Lamley et al., 2015a).

### Case 2: Model-Free Analysis of μs-Motion

Microsecond motion is the result of processes having higher free-energy cost than nanosecond and picosecond dynamics. We suggest dividing these motions into local and collective motions, where the free energy cost of local motions comes from higher amplitude motions (∼10°) that require traversing a large energy barrier. In contrast, collective motions tend to be very low amplitude motion, where the high free-energy cost of the motion is not due to large amplitude dynamics or a significant energy barrier, but rather diffusive dynamics involving large numbers of atoms. Such dynamics are characterized by modes of motion, where a continuum of possible correlation lengths leads to a distribution of correlation times. In contrast, some local microsecond dynamics can be reasonably well approximated as a hopping motion between two orientations, and therefore described with a single correlation time (although effort should be made to determine whether relaxation might be due to multi-site exchange, and understand how this changes the interpretation of data analysis).

#### Local Dynamics

The availability of *R*_1*ρ*_ data, including formulas for its analysis (Trott and Palmer, 2002; Abergel and Palmer, 2003; Miloushev and Palmer, 2005; Kurbanov et al., 2011; Rovo and Linser, 2017) and improving methods for its collection (Kurauskas et al., 2017; Lakomek et al., 2017; Keeler et al., 2018; Krushelnitsky et al., 2018) has recently resulted in considerable improvement in the ability to characterize local micro- to millisecond motions (Rovó, 2020). We consider two categories of *R*_1*ρ*_ experiments: the first is Bloch-McConnell relaxation dispersion experiments (BMRD), for which *R*_1*ρ*_ relaxation is the result of motion modulating the isotropic chemical shift, and the NEar Rotary-resonance Relaxation Dispersion (NERRD, (Kurauskas et al., 2017)), for which orientational changes in anisotropic tensors leads to *R*_1*ρ*_ relaxation. For two-site exchange, BMRD *R*_1*ρ*_ relaxation rate constants depend on exchange rate ( $k_{\text{ex}}=1/\tau_{c}$ ), the change in isotropic chemical shift due to exchange ( $\Delta \omega_{12}$ ), and the population ( $p_{1},p_{2}=1-p_{1}$ ). Rate constants further depend on the effective field strengths corresponding to each of the two chemical shifts, $\omega_{\text{e}1}$ and $\omega_{\text{e}2}$ , as well as the effective field for the average chemical shift. Palmer and coworkers provide us with the following expression (Trott and Palmer, 2002; Trott et al., 2003; Miloushev and Palmer, 2005), which is valid in the fast or intermediate exchange regimes:

$$\begin{array}{l} R_{1\rho }=\frac{R_{1}}{2}\left(1+\cos^{2}\beta_{\text{e}}\right)+R_{1\rho }^{\text{DD,CSA}}+R_{1\rho }^{\text{ex}}\\ R_{1\rho }^{\text{ex}}=\frac{\sin^{2}\beta_{\text{e}}p_{1}p_{2}\Delta \omega_{12}^{2}k_{\text{ex}}}{\frac{\omega_{\text{e}1}^{2}\omega_{\text{e}2}^{2}}{\omega_{\text{e}}^{2}}+k_{\text{ex}}^{2}-\sin^{2}\beta_{\text{e}}p_{1}p_{2}\Delta \omega_{12}^{2}\left(1+\frac{2k_{\text{ex}}^{2}(p_{1}\omega_{\text{e}1}^{2}+p_{2}\omega_{\text{e}2}^{2})}{\omega_{\text{e}1}^{2}\omega_{\text{e}2}^{2}+\omega_{\text{e}}^{2}k_{\text{ex}}^{2}}\right)}\\ \;\Omega =p_{1}\Omega_{1}+p_{2}\Omega_{2}\\ \;\omega_{\text{e}}^{2}=\omega_{1}^{2}+\Omega^{2}\\ \;\omega_{\text{e}1}^{2}=\omega_{1}^{2}+\Omega_{1}^{2},\omega_{\text{e}2}^{2}=\omega_{2}^{2}+\Omega_{2}^{2} \end{array}$$ (33)

The total $R_{1\rho }$ relaxation has contributions from longitudinal relaxation (*R*_1_), transverse relaxation from dipole and CSA tensors ( $R_{1\rho }^{\text{DD,CSA}}$ ), and from chemical exchange ( $R_{1\rho }^{\text{DD,CSA}}$ ). (Kurbanov et al., 2011) give the formula for $R_{1\rho }^{\text{DD,CSA}}$ .

$$\begin{array}{l} R_{1\rho }^{\text{DD},\text{CSA}}=\sin^{2}\beta_{e}\times [{\left(\frac{\delta }{4}\right)}^{2}\left(J\left(\omega_{\text{S}}\right)+\frac{1}{3}J\left(2\omega_{\text{r}}-\omega_{\text{e}}\right)+\frac{2}{3}J\left(\omega_{\text{r}}-\omega_{\text{e}}\right)+\frac{2}{3}J\left(\omega_{\text{r}}+\omega_{\text{e}}\right)+\frac{1}{3}J\left(2\omega_{\text{r}}+\omega_{\text{e}}\right)\right)\\ \;+\frac{2}{27}{\left(\omega_{\text{I}}\Delta \sigma_{\text{I}}\right)}^{2}\left(\frac{1}{2}J\left(2\omega_{\text{r}}-\omega_{\text{e}}\right)+J\left(\omega_{\text{r}}-\omega_{\text{e}}\right)+J\left(\omega_{\text{r}}+\omega_{\text{e}}\right)+\frac{1}{2}J\left(2\omega_{\text{r}}+\omega_{\text{e}}\right)\right)] \end{array}$$ (34)

$\omega_{\text{r}}$ is the magic angle spinning frequency, and $\omega_{\text{e}}$ is the effective field as defined above. If one assumes the microsecond dynamics are dominated by two-site hoping, the spectral density is given simply by

$$J\left(\omega \right)=\frac{2}{5}3p_{1}p_{2}\left(1-\cos^{2}\theta \right)\frac{k_{\text{ex}}}{k_{\text{ex}}^{2}+\omega^{2}}=\frac{2}{5}\left(1-S^{2}\right)\frac{\tau_{c}}{1+{\left(\omega \tau_{c}\right)}^{2}}$$ (35)

**FIGURE 12:**
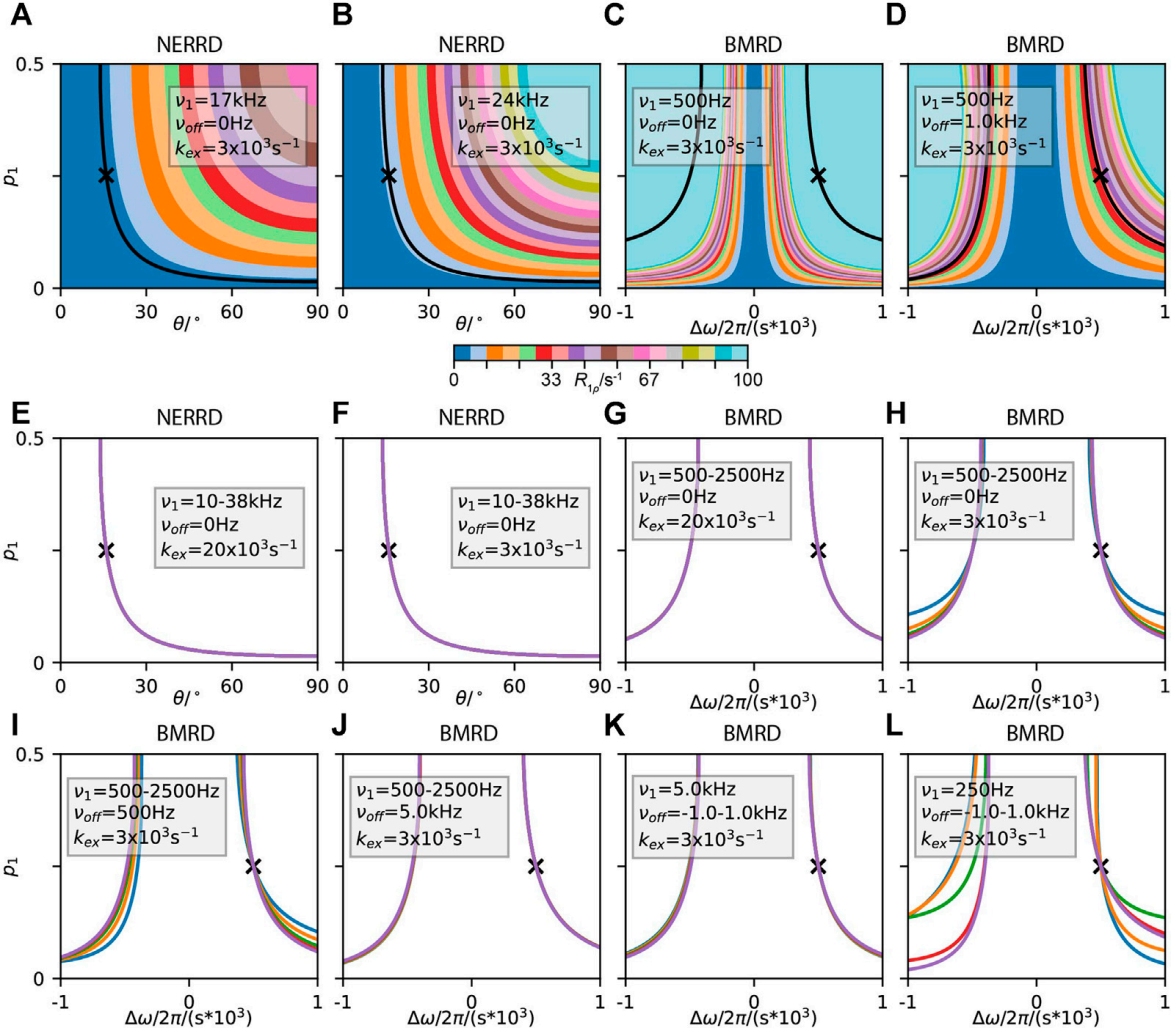
Separating population from hop angle and change in chemical shift in NERRD and BMRD experiments. Relevant parameters are shown as insets ( $\omega_{r}/2\pi$ =40 kHz for NERDD plots). In **(A–D)**, contour plots are shown for NERDD and BMRD relaxation rate constants under various conditions, and in each plot, a contour shows all values of $p_{1}$ and θ or $\Delta \omega_{1}$ that yield $R_{1\rho }$ equal to the value obtained for $p_{1}$ = 0.25 and $\theta =16^{{}^{\circ}}$ or $\Delta \omega_{\text{I}}$ = 500 Hz (marked as a cross on each plot). In **(E–L)**, we only show the contour, but for a range of experimental conditions (five experiments, linearly spaced, with range indicated in the plot). In some cases, this yields nearly identical contours, such that we only see one of the five contours.

Then, the question is, how may we most efficiently extract the exchange rate ( $k_{\text{ex}}=1/\tau_{c}$ ), populations ( $p_{1},p_{2}$ ), chemical shift changes ( $\Delta \omega_{12}$ ), and angle changes ( θ ). Fitting of $k_{\text{ex}}$ has been fairly well established using both NERDD or BMRD (Trott et al., 2003; Ma et al., 2014; Rovo and Linser, 2017; Marion et al., 2019), and combining both methods should improve the accuracy of the resulting $k_{\text{ex}}$ . However, separation of populations from either θ (NERDD) or $\Delta \omega_{12}$ (BMRD) is non-trivial. Supposing we already know $k_{\text{ex}}$ , a given experiment’s relaxation rate constant then depends on the populations and either θ or $\Delta \omega_{12}$ (at sufficiently fast MAS, a given effective field usually results in either $R_{1\rho }^{\text{DD,CSA}}$ or $R_{1\rho }^{\text{ex}}$ being dominant, although in principle both terms are active in the same experiments). Inspecting Eqs. 34, 35 we note that terms $p_{1},p_{2}$ , and θ , only appear once as a product of terms, $3p_{1}p_{1}\left(1-\cos^{2}\theta \right)$ . Then, based on NERRD data alone, these parameters are inseparable. This is seen in Figure 12, where we plot $R_{1\rho }$ as a function of $p_{1}$ and θ . We also calculate $R_{1\rho }^{\text{DD,CSA}}$ specifically for $p_{1}$ = 0.25 and $\theta =16^{{}^{\circ}}$ , and then indicate all other positions resulting in the same value of $R_{1\rho }^{\text{DD,CSA}}$ Figures 12A,B as a black contour. In Figures 12E,F, we only show contours where $R_{1\rho }^{\text{DD,CSA}}$ matches the value obtained for $p_{1}$ = 0.25 and $\theta =16^{{}^{\circ}}$ , but show several different experimental conditions (varying the field strength, $\nu_{1}=\omega_{1}/2\pi$ ). Because this results in identical contours, we are unable to disentangle these parameters based on NERRD experiments under different conditions.

In contrast, $R_{1\rho }$ relaxation resulting from chemical exchange has a more complex dependence on the various parameters. In particular, effective fields for each of the two states in exchange, $\omega_{\text{e}1}$ and $\omega_{\text{e}2}$ depend on the different offsets, $\Omega_{1}$ , $\Omega_{2}$ , but do not depend on the populations, in principle making the terms separable. Indeed, several plots in Figure 12 show that different experimental conditions lead to different contours for $p_{1}$ vs. $\Delta \omega_{12}$ (contours correspond to $R_{1\rho }$ that is equal to $R_{1\rho }$ obtained for $p_{1}$ = 0.25 and $\Delta \omega_{12}$ = 500 Hz, where contour intersections yield the input values). We are then able to identify the critical conditions required for separating population from chemical shift change. First, we see that if $k_{\text{ex}}\gg \Delta \omega_{12}$ , contours are fully overlapped so that we are not able to separate the terms, shown in Figure 12G. This is because, in Eq. 33, $k_{\text{ex}}^{2}$ must be much larger than the last term in the denominator. If it is also larger than $\omega_{\text{e}1}^{2}\omega_{\text{e}2}^{2}/\omega_{\text{e}}^{2}$ , then the critical dependence of the $R_{1\rho }$ on $\omega_{\text{e}1}$ or $\omega_{\text{e}2}$ is lost. In case $k_{\text{ex}}^{2}$ is not larger than $\omega_{\text{e}1}^{2}\omega_{\text{e}2}^{2}/\omega_{\text{e}}^{2}$ , then the effective field must be much larger than $\Delta \omega_{12}$ , so that this term converges on $\omega_{e}^{2}$ , again losing dependence on $\omega_{\text{e}1}$ and $\omega_{\text{e}2}$ (i.e. the denominator simplifies to $\omega_{\text{e}}^{2}+k_{\text{ex}}^{2}$ (Trott and Palmer, 2002)). In any case, if the effective fields become large, $\omega_{\text{e}1}\to \omega_{e}$ , $\omega_{e2}\to \omega_{e}$ , similarly preventing separation in terms. For example, see Figures 12J,K, where a large offset or large field strength on the spin-locking field results in overlapping contours. Finally, note that we require a frequency offset to be applied in order to obtain the sign of $\Delta \omega_{12}$ . If no frequency offset is applied, then all contours are symmetric as in Figure 12H.

Separability occurs only when $k_{\text{ex}}$ , $\Delta \omega_{12}$ , and $\omega_{\text{e}}$ are of similar size. Restricting $\omega_{\text{e}}$ is particularly challenging in solid-state NMR, where coherent effects may contribute to relaxation when the spin-locking field becomes too small (Öster et al., 2019). One approach would be to use increasing spinning frequencies (Penzel et al., 2015; Lakomek et al., 2017), although we note that some of the most clear improvements in Figure 12 occur in Figure 12L, where the field strength is only a few times bigger than the H–N *J*-couplings, which cannot be averaged by spinning.

In case we are in the fast exchange limit for BMRD experiments, we are left only with the terms $p_{1}p_{2}\left(1-\cos^{2}\theta \right)$ from NERRD experiments and $p_{1}p_{2}\Delta \omega_{12}^{2}$ from BMRD experiments. In this case, there is little to be done to fully separate population from the other parameters. If values of $\Delta \omega_{12}$ may be bounded, it is then possible to also bound $p_{1}p_{2}$ , and therefore one finds a restricted range for possible values of θ (the reverse approach also works). However, if we are in the range of intermediate exchange, then we may separate populations from $\Delta \omega_{12}$ , and use the result to also obtain θ (note that inclusion of NERDD data should additionally improve the accuracy of $k_{\text{ex}}$ , which in turn improves separation of $\Delta \omega_{12}$ from populations based on the BMRD data). Note that Marion et al. have recently presented similar arguments (Marion et al., 2019), although separation of terms was apparently achieved by combining NERRD and BMRD data for fairly fast exchange ( $k_{\text{ex}}$ =18,000 s^−1^, $\Delta \omega_{12}/2\pi$ = 240 Hz). While we agree that using both data sets together is beneficial, the information to separate population must come from the BMRD data and this is only possible in the intermediate exchange regime (Marion et al. calculated *R*_1*ρ*_ for a set of conditions, and via a coarse grid search, were able to find the initial conditions, however, other solutions along contours as in our Figure 12 likely were overlooked in the grid search).

A final consideration when analyzing BMRD and NERRD data is whether a two-site exchange model is reasonable. In a true two-site exchange, all moving residues should have identical exchange rates and populations, but differing $\Delta \omega_{12}$ and θ values. Then, validation of the two-site model could be achieved by independently analyzing all residues and establishing that all fits have approximately the same $p_{1}$ , $p_{2}$ , and $k_{\text{ex}}$ (or just the same $k_{\text{ex}}$ if populations cannot be determined). In case the true behavior is, for example, three-site exchange, fitting to the two-site exchange model will yield exchange rates that are a weighted average of the two non-zero eigenvalues of the 3 × 3 exchange matrix, where weighting will depend on the chemical shifts of the three sites and/or the angles sampled. In this case, it may be appropriate to apply a three-site exchange model, while jointly fitting all residues using a common set of rate constants (four to six independent parameters, depending on the model chosen). Such an approach has been demonstrated using CPMG relaxation in solution-state NMR (Korzhnev et al., 2005; Neudecker et al., 2006), with the general equations solved for CPMG (Koss et al., 2018).

#### Collective Dynamics

NERDD relaxation also appears throughout the whole protein in the absence of BMRD relaxation, depending on sample conditions, and is attributed to low amplitude rocking of the whole protein. This is observed very weakly in GB1 crystals (Krushelnitsky et al., 2018), and strongly in GB1 complexed with IgG (Lamley et al., 2015b), HET-s (218–289) (Smith et al., 2016), ubiquitin crystals with amplitude depending heavily on crystal form (Ma et al., 2015; Kurauskas et al., 2017; Lakomek et al., 2017), and SH3 (Krushelnitsky et al., 2018). The apparent global nature of this motion led all of these studies, with the exception of Lakomek et al., to fit *R*_1*ρ*_ relaxation using a slow motion with a single correlation time for all residues. In our HET-s analysis, we proposed fitting *R*_1*ρ*_ data using a global, slow correlation time, where the corresponding order parameter could vary, and additionally an offset term that would account for faster motion that could not be fully parameterized from *R*_1*ρ*_ data alone. Kurauskas et al. also followed this procedure, whereas Krushelnitsky and coworkers included explicit fitting of an additional fast motion with a distribution of correlation times. By including an offset term, and using a single correlation time globally, we again have a linear fit.

$$R_{1\rho }=\underset{\text{ns}\;\text{motions}}{\underset{︸}{R_{1\rho }^{0}}}+\left(1-S_{\text{s}}^{2}\right)\underset{\mu \text{s}\;\text{motion},\text{fixed}}{\underset{︸}{R_{1\rho }\left(\tau_{\text{s}}\right)}}$$ (36)

**FIGURE 13:**
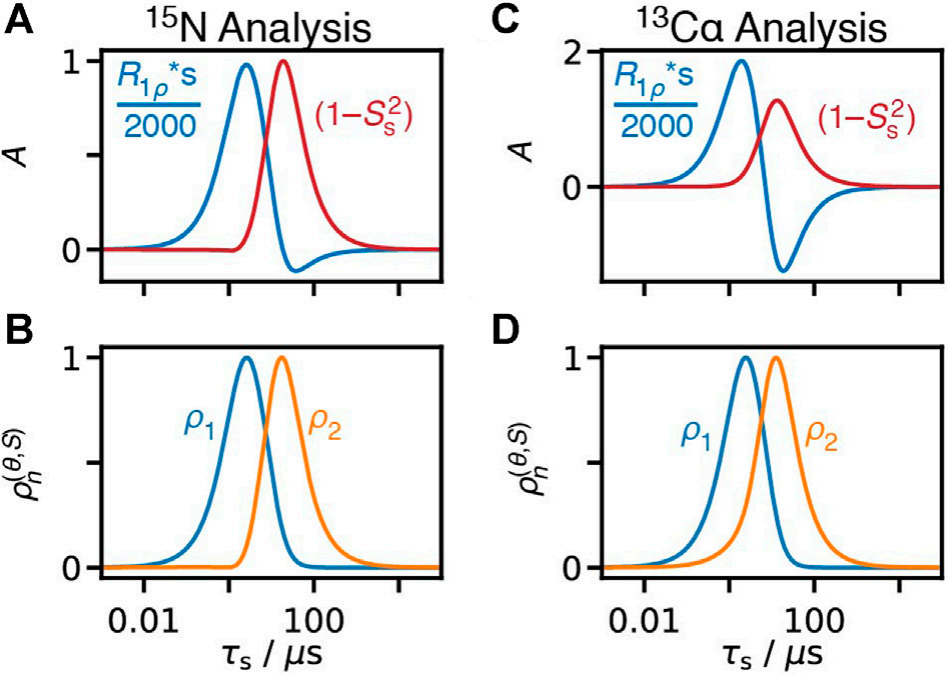
Behavior of fitting *R*_1*ρ*_ data to an offset and a fixed correlation time. **(A)** shows the offset term, $R_{1\rho }^{0}$ , divided by 2000, and the order parameter for the slow correlation time, $\left(1-S_{\text{s}}^{2}\right)$ resulting from fitting calculated relaxation rate constants as a function of correlation time to Eq. 36. Experiments are ^15^N *R*_1*ρ*_ acquired with MAS frequency of 60 kHz and spin-lock strengths of 11, 16, 25, 38, and 51 kHz, and $\tau_{\text{s}}$ is fixed at 18.5 μs. **(B)** shows detector sensitivities optimized using the same data set. **(C)** shows $R_{1\rho }^{0}$ divided by 2000 and $\left(1-S_{\text{s}}^{2}\right)$ for ^13^C *R*_1*ρ*_ acquired with MAS frequency of 60 kHz and spin-lock strengths of 9, 18, 35, and 48 kHz, as well as an additional experiment with MAS frequency of 40 kHz and spin-lock strength of 25 kHz $\tau_{\text{s}}$ is fixed at 7.0 μs. **(D)** shows detector sensitivities optimized using the same data set.

Then, for each residue, $R_{1\rho }^{0}$ and $\left(1-S_{\text{s}}^{2}\right)$ are varied, where $R_{1\rho }^{0}$ in principle compensates for relaxation due to fast, nanosecond motion, and $\left(1-S_{\text{s}}^{2}\right)$ should determine the effective amplitude of the global motion, with correlation time $\tau_{s}$ , on the given residue. Practically, what happens is that the *R*_1*ρ*_ rate constants measured for a given residue have certain ratios. If those ratios match the ratios calculated for $\tau_{\text{s}}$ , then $R_{1\rho }^{0}=0$ and the relaxation rate constants are fitted only with ( $1–S_{\text{s}}^{2}$ ). In contrast, if all rate constants are approximately equal, then $\left(1-S_{\text{s}}^{2}\right)=0$ and $R_{1\rho }^{0}$ accounts for the full relaxation. However, in most cases, the ratios are closer to one than predicted by $\tau_{\text{s}}$ , but not exactly one and so by including contributions from $R_{1\rho }^{0}$ and $\left(1-S_{\text{s}}^{2}\right)R_{1\rho }\left(\tau_{\text{s}}\right)$ , the data may be fit. One may investigate in more detail how the two terms vary as a function of correlation time (as in Figures 6–8). We show the behavior for the ^15^N and ^13^Cα *R*_1*ρ*_ data sets found in Smith et al. (2016) in Figure 13.

In Figure 13A, we calculate *R*_1*ρ*_ relaxation rate constants for ^15^N relaxation, and fit to Eq. 36. $\left(1-S_{\text{s}}^{2}\right)$ reaches a maximum of approximately one at 19 μs, so that this parameter describes motion at and around the fixed correlation time of $\tau_{\text{s}}$ = 18.5 μs. On the other hand, the offset term, $R_{1\rho }^{0}$ , actually is most sensitive at 2.5 μs, far from fitting primarily fast, nanosecond motion. We see that the functional forms are similar to detector sensitivities optimized from the same data set, Figure 13B. In Figure 13C, the behavior is less ideal: $\left(1-S_{\text{s}}^{2}\right)$ reaches a maximum of 1.28 at 13 μs, somewhat removed from the fixed correlation time of 7.0 μs, and the offset term becomes negative for correlation times around 18 μs.

The sensitivity of the offset term in Figure 13A to motion near 2.5 μs as opposed to faster motions may be surprising, although perhaps it should not be. NERRD experiments are most sensitive in the μs-range of correlation times, and rate constants under different experimental conditions have nearly converged to the same value at 1.9 μs (all rate constants within 5% of each other)– only slightly faster than the 2.5 μs where we find the maximum. Then, we would expect the offset term to be sensitive both near where *R*_1*ρ*_ is most sensitive, but also near where it converges, which is roughly what we find.

It is then important to note that fitting $R_{1\rho }$ to contributions from an offset term and a fixed correlation time results in an offset term that is most sensitive not to fast (nanosecond) motions, but rather to slower (microsecond) motions. In some cases, $\left(1-S_{\text{s}}^{2}\right)$ may be overly sensitive to some correlation times, with sensitivity exceeding one at positions that are removed from $\tau_{\text{s}}$ . Detectors are also a better choice for characterizing broad distributions of correlation times, if one does not know the form of the distribution. In fact, we suspect that global rocking motion is the result of collective dynamics over varying length scales, where increasing the correlation length also increases the correlation time, and therefore yields a broad distribution of correlation times. We demonstrated the relationship between correlation length and correlation time window for HET-s fibrils on the nanosecond timescale using a combination of NMR and MD simulation (Smith et al., 2019b), however, the question remains whether similar behavior can fully explain rocking motion of crystalline proteins; for example, Schanda and coworkers argue that a coupling between overall rocking motion and local loop motion may exist in crystalline ubiquitin (Kurauskas et al., 2017).

## Outlook: Combining Methods

We have seen that relaxation data in NMR may be processed by a variety of different methods, however, only some of these methods can really be thought of as “model-free,” such that we can establish a well-defined (linear) behavior for each parameter as a function of correlation time, independent of the actual model of the correlation function. These methods are the original model-free analysis, under the assumption that $\omega \tau_{i}\ll 1$ , spectral density mapping, LeMaster’s approach, and detector analysis. Of these, only detector analysis is generally applicable to solid-state NMR.

So, are detectors the last word in NMR dynamics analysis? We certainly hope not. Each detector response provides a “window” into the total reorientational motion of some NMR tensor, with the window width and center defined by $\rho_{n}\left(z\right)$ . Still, such a description is not very precise: a moderate detector response could result from a low amplitude motion near where $\rho_{n}\left(z\right)$ reaches its maximum, it could result from a high amplitude motion where $\rho_{n}\left(z\right)$ is small, or (and we suspect this is often the case), it characterizes a distribution of correlation times that overlaps the range of sensitivity of that detector. A collection of detectors, and their behavior as a function of position in a molecule gives further hints at the dynamics of a molecule, but leaves much to be desired in terms of details of motion. What we would rather have is better models of motion. If we use a *good* model, based on knowledge of the dynamics obtained from other methods, the information added to our experimental data should improve our interpretation of the experiment.

Molecular dynamics simulation is particularly powerful as a complimentary method to NMR. One obtains positions of all atoms as a function of time, allowing first, the direct calculation of the NMR-relevant correlation functions, and second, in principle allowing one to connect those correlation functions to specific motion in the molecule. C(t) is explicitly calculated as

$$\begin{array}{l} C\left(t_{n}\right)=\frac{1}{N}\sum_{i=0}^{N-n-1}P_{2}\left(\vec{\mu }\left(\tau_{i}\right)\cdot \vec{\mu }\left(\tau_{i+n}\right)\right)\\ \;\;\;\;\;\;\;\;\;\;\;\approx S^{2}+\left(1-S^{2}\right)\int_{-\infty }^{\infty }\theta \left(z\right)\underset{R_{C\left(t_{n}\right)}\left(z\right)}{\underset{︸}{e^{-t_{n}/\left(10^{z}\cdot 1\;\text{s}\right)}}}dz \end{array}$$ (37)

This is the discrete form of Eq. 14, as would be applied to an MD trajectory. To obtain the *n*th time point in the correlation function, $C\left(t_{n}\right)$ , we simply average over all pairs of frames separated by *n* frames. The latter equation is our assumed form for the correlation function, where we note that a given time point of the correlation function, $C\left(t_{n}\right)$ , is related to the distribution of correlation times with the same functional form as the relaxation rate constants (excepting the offset, $S^{2}$ , see Eq. 27). This allows one to calculate detectors from the collection of time points in MD-derived correlation functions using a procedure nearly identical to that described in *Optimizing Detector Sensitivities: Automated Approach*, where the sensitivity, $R_{\zeta }\left(z\right)$ (Eq. 27), is replaced by the term $R_{C\left(t_{n}\right)}\left(z\right)=\exp\left(-t_{n}/\left(10^{z}\cdot 1\;\text{s}\right)\right)$ . In fact, not only may detector analysis be easily modified to analyze MD-derived data, but it is a general approach to numerically solving the inverse Laplace transform, which avoids some of the pitfalls of more common regularization approaches (Tikhonov and Arsenin, 1977).

**FIGURE 14:**
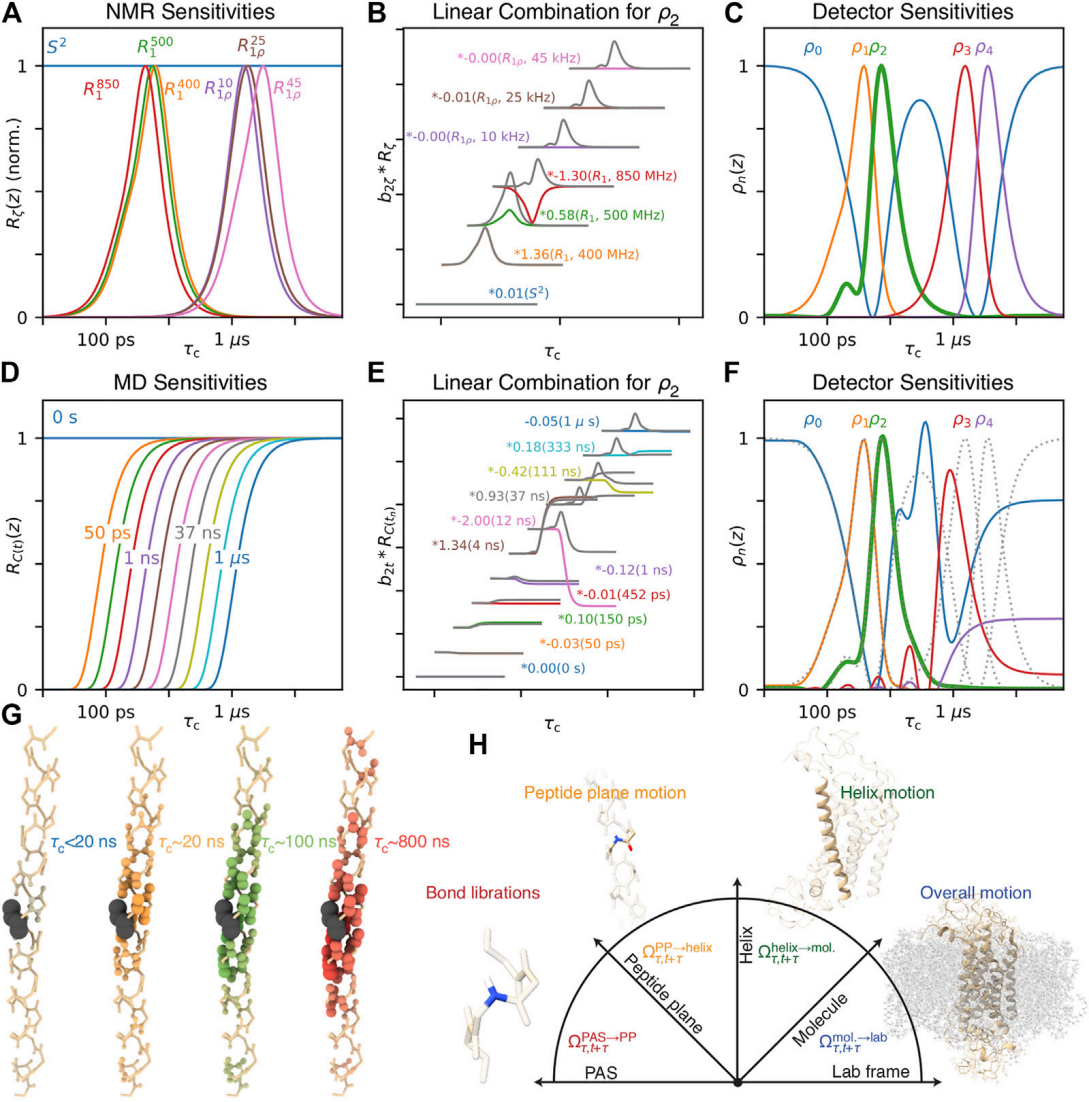
Combining NMR and MD. **(A)** plots normalized NMR sensitivities for a selection of experiments (*S* ^2^, ^15^N *R*_1_ at 400, 500, 850 MHz, ^15^N *R*_1*ρ*_ at 850 MHz, 60 kHz MAS, *ν*_1_ at 10, 25, and 45 kHz). **(B)** shows a linear combination of the normalized sensitivities (*x*, *y* positions shifted to reduce plot overlap), which yields the sensitivity of *ρ*_2_, shown in **(C)** (green, bold). In color are the weighted contributions from each rate constant, and grey shows the cumulative sum (summing all sensitivities at and below the grey line). **(C)** shows the five sensitivities optimized from NMR data. **(D)** plots sensitivities of time points from MD-derived correlation functions (0 s, 10 points log-spaced from 50 ps to 1 μs). **(E)** shows a linear combination of those sensitivities, optimized to match the sensitivity of *ρ*_2_ (*x, y* positions shifted to reduce plot overlap). **(F)** shows detectors optimized to match the NMR-derived detectors in **(C)**. **(G)** shows spatial correlation of motion in a helix as a function of correlation time (windows for <20, ∼20, ∼100, ∼800 ns). Color intensity and bond radii indicate the correlation coefficient between that residue’s H–N motion and the motion of the black residue. **(H)** illustrates frames used to separate transformation from the PAS to the lab frame into four steps: a peptide plane frame, a helix frame, and a molecule frame (illustration inspired by Brown (1996), molecule plots created with ChimeraX (Pettersen et al., 2021)).

When analyzing MD with detectors, one has two options: find the optimal set of detectors for describing correlation time distributions found with MD (that is, as many as possible with good signal-to-noise, and as narrow/non-overlapping as possible), or optimize the detectors to match some or all of the NMR-derived detectors. The latter approach is shown in Figure 14, where sensitivities of seven NMR experiments in Figure 14A are optimized to yield five detectors in Figure 14C, and the linear combination used to yield $\rho_{2}\left(z\right)$ is explicitly illustrated in Figure 14B. From MD, time points in the correlation function may also be linearly combined (sensitivities for 11 time points shown in Figure 14D), to match the NMR-derived detectors Figures 14E,F. Note that in Figure 14F, the linear combination is a very good match for *ρ*_1_ and *ρ*_2_, with moderate success for *ρ*_0_, but detector sensitivities in the microsecond range are badly reproduced. The detector optimization indicates (correctly) that a 1 μs trajectory cannot reasonably predict dynamics in the range of several microseconds (*ρ*_3_, *ρ*_4_ in red, violet), thus providing a means for determining what information can and cannot be compared across methods. Where sensitivities agree, *quantitative* comparison of dynamics in MD and NMR is possible. Note that in Figures 14D–F, we only show 11 time points for illustrative purposes, but this procedure is equally valid for ∼10^6^ time points (for such a long correlation function, calculating Eq. 37 takes much longer than evaluating its result with detectors).

The detector analysis then provides a very reliable means of comparing NMR results to MD simulation. The ability to easily compare results across multiple methods is one of the primary advantages of detector analysis. We should note that carefully executed fitting of MD-derived correlation functions, followed by calculation of relaxation rate constants should yield similarly reliable rate constants, if the trajectory is sufficiently long (Mollica et al., 2012). However, the rate constants themselves are sensitive to a broader range of correlation times than detectors, so that the comparison has lower timescale resolution than detectors.

With MD and NMR data sets, one may then use NMR data via detectors (or relaxation rate constants) as a means of validating the MD, and potentially refining it; methods include selecting sections of trajectories that best reproduce experiment (Salvi et al., 2016), selecting the best force fields for a system (Antila et al., 2021), or validating the refinement of a force-field itself (Hoffmann et al., 2018a; Hoffmann et al., 2018b). One may also use NMR data (specifically order parameters) as a means of directing the simulation, so that the simulation returns parameters matching the experiment (Hansen et al., 2014). One should note that a major challenge of combining NMR and MD data is that, while NMR is highly sensitive to microsecond motions, for example, via *R*_1*ρ*_ measurements, it is challenging to obtain accurate dynamics on the microsecond timescale from MD simulations. Although MD simulations now regularly extend for multiple microseconds, or longer via enhanced sampling (Bernardi et al., 2015), one still lacks sufficient statistics to obtain reliable dynamics behavior. Consider, if we investigate a 1 μs motion, using a 10 μs trajectory, we should observe 10 events, but the variance in number of events is also 10 (assuming Poisson statistics), so that large errors easily occur. Additionally, correct replication of slower motions requires all the faster motion leading up to the slow motion to occur at approximately the correct rates, so that the slower motions are more susceptible to influences like force field inaccuracies, starting structure of the system, etc. This remains a significant challenge for combining experiment in simulation, requiring creative solutions to take advantage of simulation where reproduction of experimental observables is poor.

With experimental validation or refinement of an MD simulation, one may analyze the simulation further, with improved confidence of the accuracy of the simulation. However, we want to use the simulation specifically to improve our interpretation of the experimental parameters. For example, we recently showed that it was possible to calculate the spatial correlation of motions within a given detector window between different residues in HET-s (218–289) fibrils (Smith et al., 2019b), using a modified iRED analysis (Prompers and Brüschweiler, 2001; Prompers and Brüschweiler, 2002). The result is that we could see that detector windows corresponding to longer correlation times tended to result in correlation over longer distances, providing at least some explanation for the presence of slow, low amplitude motion in fibrils. A similar correlation analysis is shown in Figure 14G, in this case for residues in an α-helix, where similarly, detector windows corresponding to longer correlation times yield longer correlation lengths. We suspect this behavior to be nearly universal: even in well-defined structures, there is always some residual flexibility. Then, both short- and long-range modes of motion should be thermally populated (in terms of modes, these are more accurately described as having short and long wavelengths). However, the longer-range modes usually have longer correlation times, resulting in the trends in Figure 14G. Note that this implies that there should almost always be distributions of correlation times due to varying correlation length, further complicating the interpretation of the two to three correlation times provided by the EMF approach.

For fairly rigid regions of a molecule, we expect detector-specific correlation analysis to help explain dynamic trends. However, what should we do for regions that are more mobile, with multiple types of motion contributing? Having all of the atom positions in an MD simulation should provide the detail that would allow us to separate different motions. Then, we could define the total motion of a bond as resulting from the product of these motions. For example, for an H–N dipole coupling in an α-helix, the total rotation of the dipole is the result of the reorientation of the principal axis system (PAS) of the dipole within the peptide plane (PP), the peptide plane reorienting within the helix, the helix reorienting with the molecule, and the molecule reorienting within the lab frame.

$$\begin{array}{l} \vec{v}\left(t+\tau \right)=R\left(\Omega_{\tau ,t+\tau }\right)\cdot \vec{v}\left(\tau \right)\\ \;\;\;\;\;\;\;\;\;\;\;\;\;\;\;\;\;=R\left(\Omega_{\tau ,t+\tau }^{\text{mol}\text{.}\to \text{lab}}\right)\cdot R\left(\Omega_{\tau ,t+\tau }^{\text{helix}\to \text{mol}\text{.}}\right)\cdot R\left(\Omega_{\tau ,t+\tau }^{\text{PP}\to \text{helix}}\right)\cdot R\left(\Omega_{\tau ,t+\tau }^{\text{PAS}\to \text{PP}}\right)\cdot \vec{v}\left(\tau \right) \end{array}$$ (38)

This concept is illustrated in Figure 14H. In the case that it is possible to derive a correlation function from each rotation, one then may effectively achieve an *in silico* model-free type separation of the correlation functions motion. A similar approach for the specific separation of librations, φ/ψ reorientation, and peptide plane tumbling in intrinsically disorded proteins has been demonstrated by Salvi et al. (2017), however we find that it is possible to fully generalize this concept for separation of arbitrary definitions of independent motions (manuscript under revision, (Smith et al., 2021b)). Then, separated motions may also be analyzed with detectors, to determine how both experimental and simulated detector responses depend on both timescale and position in the molecule. Separation of motions could also be coupled with mode analyses such as iRED (Prompers and Brüschweiler, 2001, 2002) or principal component analysis (Amadei et al., 1993; Altis et al., 2007), providing a method to better characterize distributions of correlation times arising from different motions and complex mode-like dynamics. In each proposed case, comparison of the different MD analyses is possible via the detector analysis. Our eventual goal is that one may extract enough detail from the MD to build explicit models of motion for direct application to the NMR experimental results, so that the final characterizations are no longer model-free at all, but rather yield highly detailed models based on the combined information from experiment and simulation.

## Conclusion

We show that the original model-free approach, SDM, LeMaster’s approach, and detectors all belong to a class of methods where fit parameters are resulting from a linear combination of experimental relaxation rate constants (potentially requiring an additional arithmetic step to yield the final parameters). IMPACT is a close approximation to this behavior, whereas EMF parameters exhibit significantly different behavior. Analysis methods belonging to this class are particularly useful because it is straightforward to estimate the resulting parameters if the distribution of correlation times, $\left(1-S^{2}\right)\theta \left(z\right)$ , is known. This is particularly advantageous when determining if a model is consistent with experimentally determined parameters, and also allows easy comparison of multiple methods.

The detector analysis is the most general of these approaches, being applicable to any collection of NMR relaxation experiments probing reorientational motion, and can be generalized for other methods such as MD simulation, requiring very little modification of the analysis. Then, the resulting detector responses from NMR and MD are easily compared. With experimental validation of MD, one may then use the wealth of detail in MD simulation to better understand how experimentally derived parameters are related to specific motion, via correlation of motion, separation of motion, and other existing and yet-to-be developed techniques. This has the potential to lead to improved models of motion for NMR analysis, which in turn can help obtain a more fundamental understand of dynamics in biomolecular systems.
