# Supplementary material for: Model-Free or Not?
Source: Front Mol Biosci. 2021 Oct 25;8:727553. doi: 10.3389/fmolb.2021.727553 (PMC8573340; doi:10.3389/fmolb.2021.727553)
Supplement: Supplementary file 1 [file DataSheet1.pdf]

Supplementary Material

S1 Calculating S^2

S^2 is defined as the limit as the correlation function approaches infinity, that is

$$S^2 = \lim_{t\to\infty} C^{\text{intern}}(t) = \lim_{t\to\infty} \left\langle P_2(\vec{\mu}(\tau)\cdot\vec{\mu}(t+\tau))\right\rangle_\tau, \qquad (1)$$

Here, $P_2(x) = (3x^2-1)/2$ is the second Legendre polynomial, and $\vec{\mu}(\tau)$ is a normalized vector that indicates the direction of the principle component of the interaction tensor at time τ. The dot product $\vec{\mu}(\tau)\cdot\vec{\mu}(t+\tau)$ then yields the cosine of the angle between the vector at times τ and $t+\tau$. When these times are infinitely far apart, they are completely uncorrelated. Thus, the probability of a given orientation at either time is given simply by the equilibrium distribution of orientations. Suppose $\vec{\mu}(\beta,\gamma)$ is the orientation corresponding to Euler angles β and γ (for a symmetric tensor, α is undefined for rotation of the tensor from its principle axis), then the probability density of that orientation is defined here as $p_{\text{eq}}(\beta,\gamma)$. Then, to obtain S^2, we must simply integrate over all possible starting orientations and all possible final orientations, weighted by probabilities $p_{\text{eq}}(\beta,\gamma)$, yielding the following integral.

$$S^2 = \int_0^\pi \sin\beta_2\, d\beta_2 \int_0^{2\pi} d\gamma_2 p_{\text{eq}}(\beta_1,\gamma_1)\int_0^\pi \sin\beta_1\, d\beta_1 \int_0^{2\pi} d\gamma_1 p_{\text{eq}}(\beta_1,\gamma_1) P_2(\vec{\mu}(\beta_1,\gamma_1)\cdot\vec{\mu}(\beta_2,\gamma_2)), \qquad (2)$$

This result may also be represented as a discrete summation over possible orientations ($p_{\text{eq}}(\vec{\mu}_i)$ is now simply a probability, not a probability density):

$$S^2 = \sum_i\sum_j p_{\text{eq}}(\vec{\mu}_i)p_{\text{eq}}(\vec{\mu}_j)P_2(\vec{\mu}_i\cdot\vec{\mu}_j) \qquad (3)$$

S2 Calculating S_r

S_r is not, by definition, the same quantity as *S*, and instead is defined as the ratio of the anisotropy of a residual tensor that has been averaged by motion, divided by the anisotropy of the rigid tensor ($S_r = \delta_{\text{resid.}}/\delta_{\text{rigid}}$) Calculation of S_r generally requires calculation of all components of the residual tensor, followed by transformation of the residual tensor into its own principle axis system (PAS),

where finally $\delta_{\text{resid.}}$ may be easily extracted and divided by δ_{rigid}. The first step, calculation of all components of the residual tensor, is achieved using the following formula.

$$A_p = \sqrt{\frac{3}{2}}\delta \frac{1}{2\pi}\int_0^{2\pi} d\gamma \int_0^{\pi} \sin\beta \, d\beta \, p_{\text{eq}}(\beta,\gamma) D_{0p}^2(0,\beta,\gamma) \tag{4}$$

We do not include a general solution for transformation back into the PAS of the residual tensor, but for the cases treated below, we will find a solution for the specific case is relatively straightforward (in its PAS, δ is determined from the 0-component of the tensor, $A_0^{\text{PAS}} = \sqrt{3/2}\delta_{\text{resid.}}$).

S3 General relationship between S^2 and S_r for symmetric motion

Given a 3-fold or higher axis of symmetry around some axis (taken here to be along *z*-axis), we find that both S^2 and S_r are functions only of the distribution over β angles, and can further show that $S^2 = S_r^2$. In general, for *N* fold symmetry, the distribution must satisfy

$$p_{\text{eq}}(\beta,\gamma + 2\pi / N) = p_{\text{eq}}(\beta,\gamma) \text{ for all } \gamma . \tag{5}$$

Then, we may calculate S_r by first obtaining the terms A_p:

$$\begin{aligned} A_p &= \sqrt{\frac{3}{2}}\delta \int_0^{\pi} \sin\beta \, d\beta \int_0^{2\pi} d\gamma \, p_{\text{eq}}(\beta,\gamma) D_{0p}^2(0,\beta,\gamma) \\ &= \sqrt{\frac{3}{2}}\delta \int_0^{\pi} \sin\beta \, d\beta \sum_{n=0}^{N-1} \int_{2\pi n/N}^{2\pi(n+1)/N} d\gamma \, p_{\text{eq}}(\beta,\gamma) d_{0p}^2(\beta) e^{-ip\gamma} \end{aligned} . \tag{6}$$

We have first broken the inner integral into *N* parts. Given that $p_{\text{eq}}(\beta,\gamma + 2\pi / N) = p_{\text{eq}}(\beta,\gamma)$, we may shift the bounds of the inner integrals to always sweep from 0 to $2\pi / N$ without needing to change the probability density. We will need to replace $\exp(-ip\gamma)$ with $\exp(-ip(\gamma + 2\pi n / N))$.

$$
\begin{aligned}
A_p &= \sqrt{\frac{3}{2}}\delta\int_0^{\pi}\sin\beta\, d\beta\sum_{n=0}^{N-1}\int_0^{2\pi n/N} d\gamma\, p_{\text{eq}}(\beta,\gamma)d_{0p}^2(\beta)e^{-ip(\gamma+2\pi n/N)} \\
&= \sqrt{\frac{3}{2}}\delta\int_0^{\pi}\sin\beta\, d\beta\int_0^{2\pi n/N} d\gamma\, p_{\text{eq}}(\beta,\gamma)d_{0p}^2(\beta)\sum_{n=0}^{N-1}e^{-ip(\gamma+2\pi n/N)} \\
&\sum_{n=0}^{N-1}e^{-ip(\gamma+2\pi n/N)} = N\delta_p \\
A_p &= \begin{cases} \sqrt{\frac{3}{2}}\delta\int_0^{\pi}\sin\beta\, d\beta\int_0^{2\pi n/N} d\gamma\, p_{\text{eq}}(\beta,\gamma)d_{0p}^2(\beta)N & p=0 \\ 0 & \text{otherwise} \end{cases}
\end{aligned}
\quad . \tag{7}
$$

The summation over n of $\exp(-ip(\gamma+2\pi n/N))$ always yields 0, unless p=0, in which case the exponential itself is one, and therefore the sum over N terms simply yields N. Therefore, only the term A_0 survives, so that this term will determine S_{r}. Then, the only remaining term that is a function of γ is the probability density itself. Integration over γ from 0 to 2π must convert $p_{\text{eq}}(\beta,\gamma)$ into the probability density as a function of β alone ($p_{\text{eq}}(\beta)$), that is

$$
\int_0^{2\pi} p_{\text{eq}}(\beta,\gamma)\, d\gamma = p_{\text{eq}}(\beta)\,. \tag{8}
$$

However, we only integrate from 0 to $2\pi n/N$, yielding $p_{\text{eq}}(\beta)/N$. This is cancelled by multiplication with N. Then, the inner integral vanishes, yielding

$$
\begin{aligned}
A_0 &= \sqrt{\frac{3}{2}}\delta\int_0^{\pi}\sin\beta\, d\beta\, p_{\text{eq}}(\beta)\frac{3\cos^2\beta-1}{2} \\
S_{\text{r}} &= A_0/(\sqrt{3/2}\delta) = -\frac{1}{2}+\frac{3}{2}\int_0^{\pi}\sin\beta\, d\beta\, p_{\text{eq}}(\beta)\cos^2\beta
\end{aligned}
\quad . \tag{9}
$$

Further simplification is not possible without an explicit form of the distribution over β.

For comparison, we also evaluate S^2.

$$
\begin{aligned}
S^2 &= \int_0^{\pi} \sin\beta_2\, d\beta_2 \int_0^{\pi} \sin\beta_1\, d\beta_1 \int_0^{2\pi} d\gamma_2 \int_0^{2\pi} d\gamma_1 p_{\mathrm{eq}}(\beta_2,\gamma_2) p_{\mathrm{eq}}(\beta_1,\gamma_1) P_2(\vec{\mu}(\beta_1,\gamma_1)\cdot\vec{\mu}(\beta_2,\gamma_2)) \\
&= \int_0^{\pi} \sin\beta_2\, d\beta_2 \int_0^{\pi} \sin\beta_1\, d\beta_1 \sum_{m=0}^{N-1}\sum_{n=0}^{N-1} \int_{2\pi m/N}^{2\pi(m+1)/N} d\gamma_2 \int_{2\pi n/N}^{2\pi(n+1)/N} d\gamma_1 p_{\mathrm{eq}}(\beta_2,\gamma_2) p_{\mathrm{eq}}(\beta_1,\gamma_1) \\
&\qquad P_2\left(\underbrace{s_{\beta1}s_{\beta1}c_{\gamma1}c_{\gamma1} + s_{\beta1}s_{\beta1}s_{\gamma1}s_{\gamma1}}_{=s_{\beta1}s_{\beta2}\cos(\gamma_1-\gamma_2)} + c_{\beta1}c_{\beta1} \right)
\end{aligned} \tag{10}
$$

Due to symmetry of the probability density over γ_1 and γ_2, again we may adjust its integration bounds to always run from 0 to $2\pi / N$, although we need to offset the change in integration bounds within the term $\cos(\gamma_1 - \gamma_2)$ (symmetry allows us to replace $p_{\mathrm{eq}}(\beta,\gamma + 2\pi n / N) = p_{\mathrm{eq}}(\beta,\gamma)$). Then, we may expand $P_2(x) = (3x^2 - 1)/2$, and extract all terms not depending on γ_1,γ_2 (thus integrating over γ_1,γ_2, eliminating this integral for some terms).

$$
\begin{aligned}
S^2 &= \int_0^{\pi} \sin\beta_2\, d\beta_2 \int_0^{\pi} \sin\beta_1\, d\beta_1 \sum_{m=0}^{N-1}\sum_{n=0}^{N-1} \int_{2\pi m/N}^{2\pi(m+1)/N} d\gamma_2 \int_0^{2\pi/N} d\gamma_1 p_{\mathrm{eq}}(\beta_2,\gamma_2) p_{\mathrm{eq}}(\beta_1,\gamma_1) \\
&\qquad P_2\left(s_{\beta1}s_{\beta2}\cos((\gamma_1 + 2\pi n / N) - \gamma_2) + c_{\beta1}c_{\beta1}\right) \\
&= \int_0^{\pi} \sin\beta_2\, d\beta_2 \int_0^{\pi} \sin\beta_1\, d\beta_1 \sum_{m=0}^{N-1}\sum_{n=0}^{N-1} \int_{2\pi m/N}^{2\pi(m+1)/N} d\gamma_2 \int_0^{2\pi/N} d\gamma_1 p_{\mathrm{eq}}(\beta_2,\gamma_2) p_{\mathrm{eq}}(\beta_1,\gamma_1) \\
&\qquad \frac{1}{2}\left[3\left(s_{\beta1}s_{\beta2}\cos((\gamma_1 + 2\pi n / N) - \gamma_2) + c_{\beta1}c_{\beta1}\right)^2 - 1 \right] \\
&= -\frac{1}{2} + \frac{3}{2}\int_0^{\pi} \sin\beta_2\, d\beta_2 \int_0^{\pi} \sin\beta_1\, d\beta_1 p_{\mathrm{eq}}(\beta_2) p_{\mathrm{eq}}(\beta_1) \cos\beta_1^2 \cos\beta_2^2 \\
&\quad \int_0^{\pi} \sin\beta_2\, d\beta_2 \int_0^{\pi} \sin\beta_1\, d\beta_1 \sum_{m=0}^{N-1} \int_{2\pi m/N}^{2\pi(m+1)/N} d\gamma_2 \int_0^{2\pi/N} d\gamma_1 p_{\mathrm{eq}}(\beta_2,\gamma_2) p_{\mathrm{eq}}(\beta_1,\gamma_1) \\
&\qquad \sum_{n=0}^{N-1} \frac{3}{2}\left[s_{\beta1}^2 s_{\beta2}^2 \cos^2((\gamma_1 + 2\pi n / N) - \gamma_2) + c_{\beta1}c_{\beta1}s_{\beta1}s_{\beta2}\cos((\gamma_1 + 2\pi n / N) - \gamma_2) \right]
\end{aligned} \tag{11}
$$

With 3 fold or higher symmetry, the sum over the cosine term yields zero, and the cosine-squared term yields *N/2*:

$$\sum_{n=0}^{N-1}\cos((\gamma_1+2\pi n/N)-\gamma_2)=0$$
$$\sum_{n=0}^{N-1}\cos^2((\gamma_1+2\pi n/N)-\gamma_2)=\frac{1}{2}\sum_{n=0}^{N-1}1+\cos\left(2((\gamma_1+2\pi n/N)-\gamma_2)\right)=N/2$$
$$\begin{aligned}S^2 &= -\frac{1}{2}+\frac{3}{2}\int_0^\pi \sin\beta_2\, d\beta_2 \int_0^\pi \sin\beta_1\, d\beta_1 p_{\text{eq}}(\beta_2)p_{\text{eq}}(\beta_1)\cos\beta_1^2\cos\beta_2^2 \\ &\quad +\frac{3}{2}\int_0^\pi \sin\beta_2\, d\beta_2 \int_0^\pi \sin\beta_1\, d\beta_1 \sum_{m=0}^{N-1}\int_{2\pi m/N}^{2\pi(m+1)/N} d\gamma_2 \int_0^{2\pi/N} d\gamma_1 p_{\text{eq}}(\beta_2,\gamma_2)p_{\text{eq}}(\beta_1,\gamma_1)\frac{N}{2}\sin^2\beta_1\sin^2\beta_2 \\ &= -\frac{1}{2}+\frac{3}{2}\int_0^\pi \sin\beta_2\, d\beta_2 \int_0^\pi \sin\beta_1\, d\beta_1 p_{\text{eq}}(\beta_2)p_{\text{eq}}(\beta_1)\cos\beta_1^2\cos\beta_2^2 \\ &\quad +\frac{3}{4}\int_0^\pi \sin\beta_2\, d\beta_2 \int_0^\pi \sin\beta_1\, d\beta_1 p_{\text{eq}}(\beta_2)p_{\text{eq}}(\beta_1)\sin^2\beta_1\sin^2\beta_2\end{aligned} \tag{12}$$

The integrals over γ_1, γ_2 each yield $1/N$, which is canceled either by the term $N/2$ or the summation over N. The two double integrals are each separable into the product of two equal terms, yielding

$$\begin{aligned}S^2 &= -\frac{1}{2}+\frac{3}{2}\left[\int_0^\pi \sin\beta\, d\beta p_{\text{eq}}(\beta)\cos^2\beta\right]^2+\frac{3}{4}\left[\int_0^\pi \sin\beta\, d\beta p_{\text{eq}}(\beta)\sin^2\beta\right]^2 \\ &= -\frac{1}{2}+\frac{3}{2}\left[\int_0^\pi \sin\beta\, d\beta p_{\text{eq}}(\beta)\cos^2\beta\right]^2+\frac{3}{4}\left[\int_0^\pi \sin\beta\, d\beta p_{\text{eq}}(\beta)(1-\cos^2\beta)\right]^2 \\ &= \frac{9}{4}\left[\int_0^\pi \sin\beta\, d\beta p_{\text{eq}}(\beta)\cos^2\beta\right]^2-\frac{3}{2}\int_0^\pi \sin\beta\, d\beta p_{\text{eq}}(\beta)\cos^2\beta+\frac{1}{4} \\ &= \left[-\frac{1}{2}+\frac{3}{2}\int_0^\pi \sin\beta\, d\beta p_{\text{eq}}(\beta)\cos^2\beta\right]^2 \\ &= S_{\text{r}}^2\end{aligned} \tag{13}$$

Then, we see generally that given a 3-fold or higher axis of symmetry, we obtain $S^2=S_{\text{r}}^2$, and furthermore we may obtain these values simply from the distribution over β.

S4 Obtain S for Explicit Models

S4.1 Wobbling on a cone

Wobbling on a cone is described by a distribution with a single β angle and a uniform distribution over γ, such that

$$
\begin{aligned}
&p_{\text{eq}}^{\text{woc}}(\beta,\gamma)=\frac{1}{2\pi\sin\beta}\delta(\beta-\beta^{\text{woc}})\\
&p_{\text{eq}}^{\text{woc}}(\beta)=\frac{\delta(\beta-\beta^{\text{woc}})}{\sin\beta}\\
&S=-\frac{1}{2}+\frac{3}{2}\int_0^{\pi}\sin\beta\, d\beta\frac{\delta(\beta-\beta^{\text{woc}})}{\sin\beta}\cos^2\beta\\
&=-\frac{1}{2}+\frac{3}{2}\cos^2\beta^{\text{woc}}
\end{aligned}
\tag{14}
$$

S4.2 Wobbling in a cone

The wobbling in a cone distribution is a uniform distribution of β angles, for all angles less than some maximum angle, β^{wic}. Then, we may calculate S^2 as

$$
\begin{aligned}
&p_{\text{eq}}^{\text{wic}}(\beta,\gamma)=\begin{cases}\dfrac{1}{2\pi(1-\cos\beta^{\text{wic}})} & \text{if}\,\beta<\beta^{\text{wic}}\\ 0 & \text{otherwise}\end{cases}\\
&p_{\text{eq}}^{\text{wic}}(\beta)=\begin{cases}\dfrac{1}{(1-\cos\beta^{\text{wic}})} & \text{if}\,\beta<\beta^{\text{wic}}\\ 0 & \text{otherwise}\end{cases}\\
&S=-\frac{1}{2}+\frac{3}{2}\frac{1}{(1-\cos\beta^{\text{wic}})}\int_0^{\beta^{\text{wic}}}\sin\beta\, d\beta\cos^2\beta\\
&=-\frac{1}{2}+\frac{3}{2}\frac{1}{(1-\cos\beta^{\text{wic}})}\left(-\frac{1}{3}\right)\cos^3\beta\Big|_0^{\beta^{\text{wic}}}\\
&=-\frac{1}{2}+\frac{1}{2}\frac{1-\cos^2\beta^{\text{wic}}}{1-\cos\beta^{\text{wic}}}=-\frac{1}{2}+\frac{1}{2}\frac{(1-\cos\beta^{\text{wic}})(1+\cos\beta^{\text{wic}}+\cos^2\beta^{\text{wic}})}{1-\cos\beta^{\text{wic}}}\\
&=\frac{1}{2}\cos\beta^{\text{wic}}(1+\cos\beta^{\text{wic}})
\end{aligned}
\tag{15}
$$

S4.3 Two-site hopping

Two-site hopping differs considerably from the examples so far, since it does not have a 3-fold symmetry axis. Therefore, S^2 and S_{r} are not easily related. We begin by defining the distribution, which allows for different populations of the two sites, followed by calculation of S^2. Here, $\delta(x)$ is the Dirac delta.

$$
\begin{aligned}
&p_{\mathrm{eq}}^{\mathrm{hop}}(\beta,\gamma)=\left(p_1\delta(\beta)+(1-p_1)\delta(\beta-\beta^{\mathrm{hop}})\right)\delta(\gamma)\\
&S^2=\int_0^{2\pi}d\gamma_2\int_0^{\pi}\sin\beta_2\,d\beta_2 p_{\mathrm{eq}}^{model}(\beta_1,\gamma_1)\int_0^{2\pi}d\gamma_1\int_0^{\pi}\sin\beta_1\,d\beta_1 p_{\mathrm{eq}}(\beta_1,\gamma_1)P_2(\vec{\mu}(\beta_1,\gamma_1)\cdot\vec{\mu}(\beta_2,\gamma_2))\\
&=\underbrace{\left(p_1^2+(1-p_1)^2\right)P_2(0)}_{\text{initial/final state same}}+\underbrace{2p_1(1-p_1)P_2(\cos\beta^{\mathrm{hop}})}_{\text{initial/final state different}}\\
&=2p_1^2-2p_1+1-\frac{1}{2}\left(2p_1-p_1^2\right)+3p_1\left(1-p_1\right)\cos^2\beta^{\mathrm{hop}}\\
&=1+3p_1(p_1-1)+3p_1\left(1-p_1\right)\cos^2\beta^{\mathrm{hop}}\\
&=1+3p_1(1-p_1)(\cos^2\beta^{\mathrm{hop}}-1)
\end{aligned}
\tag{16}
$$

For equal populations ($p_1=1-p_1=0.5$), this reduces to the more familiar $\frac{1}{4}+\frac{3}{4}\cos^2\beta^{\mathrm{hop}}$. We may also solve for S_{r}, beginning with a solution for all terms A_p.

$$
\begin{aligned}
&A_p/(\sqrt{3/2}\delta)=\int_0^{\pi}\sin\beta\,d\beta\sum_{n=0}^{N-1}\int_0^{2\pi n/N}d\gamma\,p_{\mathrm{eq}}(\beta,\gamma)d_{0p}^2(\beta)e^{-ip(\gamma+2\pi n/N)}\\
&=p_1\underbrace{d_{0p}^2(0)}_{=\delta_p}+(1-p_1)d_{0p}^2(\beta^{\mathrm{hop}})
\end{aligned}
\quad . \tag{17}
$$

Due to the lack of symmetry, the terms A_p for $p\neq 0$ do not vanish. Thus, we must find a rotation that brings the terms representing the residual tensor back into its own frame. For the residual tensor to be in its own frame, the terms $A_{\pm1}$ must be zero, and furthermore, $A_{\pm2}=-\delta\eta/2$ cannot have magnitude larger than $A_0/\sqrt{6}=\left(\sqrt{3/2}\delta\right)/\sqrt{6}=\delta/2$ since η may not exceed 1. This prevents the absolute value of S_r from falling below 0.5. We first attempt this with a rotation around y, using β^0.

$$
\begin{aligned}
&0 = A_1^{\mathrm{PAS}} / (\sqrt{3/2}\delta) = \sum_{q=-2}^{2} d_{q1}^{2}(-\beta^{0})\left(p_1 d_{0,q}^{2}(0) + (1-p_1) d_{0q}^{2}(\beta^{\mathrm{hop}})\right) \\
&0 = p_1 d_{01}^{2}(-\beta^{0}) + (1-p_1)\underbrace{\sum_{q=-2}^{2} d_{0q}^{2}(\beta^{\mathrm{hop}}) d_{q1}^{2}(-\beta^{0})}_{d_{01}^{2}(\beta^{\mathrm{hop}}-\beta^{0})} \\
&0 = p_1 \sqrt{\frac{3}{8}}\sin(-2\beta^{0}) + (1-p_1)\sqrt{\frac{3}{8}}\sin\left(2(\beta^{\mathrm{hop}}-\beta^{0})\right) \\
&\frac{p_1}{p_1 - 1} = \frac{\sin\left(2(\beta^{\mathrm{hop}}-\beta^{0})\right)}{\sin(2\beta^{0})} \\
&\frac{p_1}{p_1 - 1} = \frac{\sin(2\beta^{\mathrm{hop}})\cos(2\beta^{0}) - \cos(2\beta^{\mathrm{hop}})\sin(2\beta^{0})}{\sin(2\beta^{0})} \\
&\frac{p_1}{1 - p_1} + \cos(2\beta^{\mathrm{hop}}) = \cot(2\beta^{0})\sin(2\beta^{\mathrm{hop}}) \\
&\cot(2\beta^{0}) = \frac{p_1 + (1-p_1)\cos(2\beta^{\mathrm{hop}})}{(1-p_1)\sin(2\beta^{\mathrm{hop}})} \\
&\beta^{0} = \arctan\left(\frac{(1-p_1)\sin(2\beta^{\mathrm{hop}})}{p_1 + (1-p_1)\cos(2\beta^{\mathrm{hop}})}\right)/2
\end{aligned}
\quad . \qquad (18)
$$

Then, we may insert to solve for A_0^{PAS}.

$$
\begin{aligned}
&S_{\mathrm{r}} = A_0^{\mathrm{PAS}} / (\sqrt{3/2}\delta) = \sum_{q=-2}^{2} d_{q0}^{2}(-\beta^{0})\left(p_1 d_{0q}^{2}(0) + (1-p_1) d_{0q}^{2}(\beta^{\mathrm{hop}})\right) \\
&S_{\mathrm{r}} = p_1 d_{00}^{2}(-\beta^{0}) + (1-p_1)\underbrace{\sum_{q=-2}^{2} d_{0q}^{2}(\beta^{\mathrm{hop}}) d_{q0}^{2}(-\beta^{0})}_{d_{00}^{2}(\beta^{\mathrm{hop}}-\beta^{0})} \\
&S_{\mathrm{r}} = p_1\left(\frac{3\cos^2\beta^{0} - 1}{2}\right) + (1-p_1)\left(\frac{3\cos^2(\beta^{\mathrm{hop}}-\beta^{0}) - 1}{2}\right)
\end{aligned}
\quad . \qquad (19)
$$

However, this result is only correct if the formula yields $S_r \geq 0.5$, otherwise $S_{\mathrm{r}} = -0.5$. Clearly, then, S^2 and S_{r}^2 are not equal except for special cases.

S5 Extracting the correlation function from an exchange matrix

In order to generate Figure 2, we need to know what correlation times arise, and need to obtain their corresponding amplitudes. We outline the procedure here, noting that we then discretize the continuous models (wobbling-on-a-cone, wobbling-in-a-cone), to estimate the distribution of correlation times.

Suppose we have an exchange matrix, $\mathbf{k}_{\text{ex}}$, whose elements express the rate constants for exchange between various pairs of Euler angles ($\Omega = \{0,\beta,\gamma\}$).

$$\frac{d}{dt}\vec{s} = \mathbf{k}_{\text{ex}} \cdot \vec{s} \,. \tag{20}$$

One may find the equilibrium state of the matrix, simply by solving

$$\mathbf{k}_{\text{ex}} \cdot \vec{s}_{\text{eq}} = 0 \,. \tag{21}$$

Note that $\vec{s}_{\text{eq}}$ should be normalized to sum to 1. Then, each element of $\vec{s}_{\text{eq}}$ (equilibrium for the exchange matrix, $\mathbf{k}_{\text{ex}}$) corresponds to a set of Euler angles. We may therefore use $\vec{s}_{\text{eq}}$ to determine how each combination of possible starting states and ending states contributes to S^2:

$$S^2 = \sum_p \sum_q [\vec{s}_{\text{eq}}]_p [\vec{s}_{\text{eq}}]_q P_2(\vec{\mu}_p \cdot \vec{\mu}_q) \,. \tag{22}$$

Furthermore, the eigenvalues of the matrix $\mathbf{k}_{\text{ex}}$ yield the decay rates of the corresponding correlation function, such that

$$\begin{aligned} &\mathbf{k}_{\text{ex}} \vec{\varphi}_m = \lambda_m \vec{\varphi}_m \\ &C(t) = S^2 + (1-S^2)\sum_{m=1} A_m \exp(-t/\tau_m) \,. \\ &\tau_m = -1/\lambda_m \end{aligned} \tag{23}$$

Note that one of the eigenvalues is always zero, here we index such that $\lambda_0 = 0$, and the remaining eigenvalues are all negative. The zero eigenvalue is omitted from the summation, since it corresponds to S^2 ($\vec{s}_{\text{eq}} \propto \vec{\varphi}_0$). Then, to fully determine the correlation function, we must find the amplitudes, $(1-S^2)A_m$, corresponding to each of the λ_m.

We begin by determining how a system, starting in some state $\vec{s}(0)$ evolves. Using the eigenvalues and eigenvectors of the exchange matrix, we may express $\vec{s}(t)$ as a sum of time dependent amplitudes, $a_m(t)$, multiplied by the corresponding eigenvectors.

$$\begin{aligned}\vec{s}(t) &= \sum_m a_m(t)\vec{\varphi}_m = \sum_m \sum_p a_{mp}[\vec{s}(t)]_p \\ a_{mp} &= [\vec{\varphi}_0, \vec{\varphi}_1, \ldots]^{-1}_{mp}\end{aligned} \quad . \tag{24}$$

Note that the terms in the summation, $a_m(t)$, are found by computing the inverse of the matrix of eigenvectors, and multiplying $\vec{s}(t)$ by it. Inserting this result into differential equation for evolution of $\vec{s}(t)$, we obtain

$$\begin{aligned}\frac{d}{dt}\vec{s}(t) &= \mathbf{k}_{\mathrm{ex}} \cdot \vec{s}(t) \\ \frac{d}{dt}\sum_m a_m(t)\vec{\varphi}_m &= \mathbf{k}_{\mathrm{ex}} \cdot \sum_m a_m(t)\vec{\varphi}_m \\ \sum_m \frac{d}{dt}[a_m(t)]\vec{\varphi}_m &= \cdot \sum_m a_m(t)\lambda_m \vec{\varphi}_m\end{aligned} \quad . \tag{25}$$

We may then solve each term of the sums separately. This yields simple exponential time dependence. Summing together the result, we find:

$$\begin{aligned}\frac{d}{dt}a_m(t) &= \lambda_m a_m(t) \\ a_m(t) &= a_m(0)\exp(\lambda_m t) \\ \vec{s}(t) &= \sum_m a_m(0)\exp(\lambda_m t)\vec{\varphi}_m \\ \vec{s}(t) &= \sum_m \sum_p a_{mp}\exp(\lambda_m t)[\vec{s}(t)]_p \vec{\varphi}_m \\ [\vec{s}(t)]_q &= \sum_m \sum_p a_{mp}\exp(\lambda_m t)[\vec{s}(t)]_p [\vec{\varphi}_m]_q\end{aligned} \quad . \tag{26}$$

Then, to determine how the total amplitude of the correlation function results from the decay of an individual eigenstates, $\vec{\varphi}_m$, we start in state *p*, with probability $[\vec{s}_{\mathrm{eq}}]_p$, and end in state *q*, by going through the eigenstate $\vec{\varphi}_m$. The contribution to the total amplitude from these pairs of states is given by $P_2(\vec{\mu}_p \cdot \vec{\mu}_q)$. Then, we sum over all possible starting and ending states (*p* and *q*, respectively), but only traverse from *p* to *q* via eigenstate *m*.

$$
\begin{aligned}
A_m(1-S^2) &= \sum_q \sum_p a_{mp}[\vec{\varphi}_m]_q [\vec{s}_{\mathrm{eq}}]_p P_2(\vec{\mu}_p \cdot \vec{\mu}_q) \\
&= \sum_q \sum_p a_{mp}[\vec{\varphi}_m]_q [\vec{s}_{\mathrm{eq}}]_p P_2(\vec{\mu}_p \cdot \vec{\mu}_q) \qquad . \\
A_m(1-S^2) &= \sum_q \sum_p a_{mp}[\vec{\varphi}_m]_q [\vec{s}_{\mathrm{eq}}]_p P_2(\vec{\mu}_p \cdot \vec{\mu}_q)
\end{aligned} \tag{27}
$$

The resulting amplitudes may be inserted into eq. (25). To validate our result, we may sum over all *m* (including the *m*=0 term), where we should find that the total amplitude always sums to 1.

$$
\begin{aligned}
&\underbrace{S^2}_{m=0} + \sum_{m=1} A_m(1-S^2) = \sum_{m=0} \sum_q \sum_p a_{mp}[\vec{\varphi}_m]_q [\vec{s}_{\mathrm{eq}}]_p P_2(\vec{\mu}_p \cdot \vec{\mu}_q) \\
&= \sum_q \sum_p \underbrace{\sum_{m=0} a_{mp}[\vec{\varphi}_m]_q}_{\delta_{p-q}} [\vec{s}_{\mathrm{eq}}]_p P_2(\vec{\mu}_p \cdot \vec{\mu}_q) \qquad . \\
&= \sum_q [\vec{s}_{\mathrm{eq}}]_q \underbrace{P_2(\vec{\mu}_q \cdot \vec{\mu}_q)}_{=1} = \sum_q [\vec{s}_{\mathrm{eq}}]_q = 1
\end{aligned} \tag{28}
$$

The a_{mp} come from the inverse of the matrix of eigenvectors, $[\vec{\varphi}_0, \vec{\varphi}_1, ...]^{-1}$, such that the sum $\Sigma_m a_{mp}[\vec{\varphi}_m]_q$ is one if *p*=*q* but 0 otherwise (yielding the Kronecker delta, δ_{p-q}). Then, the second Legendre polynomial is only evaluated for $\vec{\mu}_q \cdot \vec{\mu}_q = 1$, thus always yielding one. Finally, only a sum over the equilibrium populations remain, which itself must sum to 1, validating our expression for the contribution of eigenstate $\vec{\varphi}_m$ to the total amplitude of the correlation function.
